# Supplementary material for: Low-power scalable multilayer optoelectronic neural networks enabled with incoherent light
Source: Nat Commun. 2024 Dec 18;15:10692. doi: 10.1038/s41467-024-55139-4 (PMC11655539; doi:10.1038/s41467-024-55139-4)
Supplement: Supplementary file 1 — Supplementary Information [file 41467_2024_55139_MOESM1_ESM.pdf]

Supplementary Information for: Low-power scalable multilayer optoelectronic neural networks enabled with incoherent light

Alexander Song[1,2*], Sai Nikhilesh Murty Kottapalli[1,2*], Rahul Goyal[1,2], Bernhard Schölkopf[3,4], Peer Fischer[1,2,5,6]

[1]Max Planck Institute for Medical Research, Heidelberg, Germany

[2]Institute for Molecular Systems Engineering and Advanced Materials, Universität Heidelberg, Heidelberg, Germany

[3]Max Planck Institute for Intelligent Systems, Tübingen, Germany

[4]ELLIS Institute Tübingen, Germany

[5]Center for Nanomedicine, Institute for Basic Science (IBS), Seoul 03722, Republic of Korea

[6]Department of Nano Biomedical Engineering (NanoBME), Advanced Science Institute, Yonsei University, Seoul, 03722, Republic of Korea

[*]Equal contribution

Supplementary Note 1: Experimental optical crosstalk and losses

Optical crosstalk in our experimental setup can be assessed by a geometric approach (see SUPP. NOTE 2 for scaled-up system including effects of diffraction). The schematic of ray-tracing through the optical layer describes the relationship between parameters and crosstalk (SUPP. FIG. 2).

We choose parameters for the LED die size, LED spacing, PD active area, PD spacing, and M to minimize crosstalk between the LED and PD pairs and the weights (see SUPP. TABLE 3 for details). Optical propagation progresses from the LED layer to a weight layer (d_1) and from the weight layer to the PD layer (d_2). We define a system magnification $M = (d_1 + d_2)/d_1$ which describes the displacement and scaling of a primary ray. The spot size from a LED of width w_{LED} propagating through a square aperture of size w_{amp} to the photodiode plane is the convolution of two squares of size $(M-1)w_{LED}$ and Mw_{amp}. The maximum extent of the spread is the sum of the sides, or $M(w_{LED} + w_{amp}) - w_{LED}$ and the center region of size $M(w_{amp} - w_{LED}) + w_{LED}$ has uniform intensity. The weights are positioned such that the center spot of each weight is exactly mapped to a single LED-PD pair.

To prevent any crosstalk between adjacent weights, the maximum spread from a LED-weight pair cannot overlap the active surface of an adjacent PD of width w_{PD}, or we require $(M(w_{LED} + w_{amp}) - w_{LED})/2 + w_{PD}/2 \leq s_{PD}$, where s stands for size.

For an optical layer of size $n \times n$ to size $m \times m$, the width of the LED array is $n \cdot s_{LED}$ and the width of the PD array is $m \cdot s_{PD}$. With a magnification M the size of the mask associated with a single LED is $m \cdot s_{PD}/M$. This results in two constraints: $s_{LED} \geq \frac{m \cdot s_{PD}}{M-1}$, which limits the minimum size of the LED array to $\frac{n \cdot m \cdot s_{PD}}{M-1}$, and the weight spacing $s_{amp} = s_{PD}/M$. Generally we aim to maximize the size of the PD active area and minimize the magnification in order to maximize the energy efficiency of the approach. This results in aiming to use as small of an LED die size as possible and minimizing s_{LED}.

Total optical losses in our experimental setup depend on both our optical parameters, choice of components, and the implemented network. While increased w_{PD} or decreased w_{amp} reduces optical crosstalk, these changes also increase collection efficiency. Similarly, while changes to d_1, d_2 have a complicated relationship with crosstalk and optical signal (see SUPP. NOTE 3), increases to the propagation distance generally reduce the amount of total light collected. For a discussion of the effects of component choice on efficiency, see SUPP. NOTE 2. The implemented network (averaged weights in the trained network were approximately 0.5, and averaged differenced weights in the network had a value of approximately 0.1).

Supplementary Note 2: Electronic circuit

The circuits implemented on the printed circuit boards (PCBs) that comprise the electronic part of the optoelectronic neural network are shown in SUPP. FIG. 5. The optoelectronic implementation consists of three circuits: one for the source, one for intermediate processing, and one for detection.

The source circuit, as depicted in SUPP. FIG. 5b, receives a 64-channel input signal from a digital-to-analog converter (DAC) and converts this voltage signal to drive the 64 LED outputs arranged in an 8×8 grid. The signal from the input board, after optical matrix-vector multiplication, is incident on a well-positioned intermediate board, shown in SUPP. FIG. 5b.

The intermediate board consists of balanced photodetection, whose signal is then amplified before being converted to a current signal to drive the LED that serves as the source for the next layer of the intermediate board.

A detector PCB is positioned at the end of the optical train to collect the light after processing through all the neural network layers. The output PCB consists of an array of photodetectors, spatially arranged as a matrix to retain the spatial information of the signal. The detected signal is then converted to a voltage signal and digitized using an analog-to-digital converter (ADC).

The parts used in the electronic circuit are tabulated in SUPP. TABLE 4

Supplementary Note 3: Analytical optical propagation solution

We approximate an analytical solution to the spread of light from a point source through our optical layers using optical parameters given in SUPP. TABLE 5. Here we describe the spread of light from a point source at $x''_0 = 0, y''_0 = 0$ that passes through a Gaussian aperture at x'_1, y'_1 on a plane d_1 away, and onto the plane (x, y). In this solution, we describe our propagation in a rotated basis where the propagation axis is defined along the vector between the source position and the aperture position.

The rotation in propagation axis results in three effective changes to our optical parameters, all determined by the scaling factor η. 1. The total distances between the source, aperture, and PD plane are scaled to Δz_1,Δz_2. 2. The Gaussian aperture is scaled in the direction parallel to the x, y displacement of the aperture by $1/\sqrt{\eta}$. 3. The output PD has to be projected back to the original PD plane, with a scaling η in the direction parallel to the displacement. Without loss of generality, for this calculation we consider the case where the displacement of the weight x'_1 is set to be the direction parallel to the propagation and y'_1 is the orthogonal direction.

With this rotation, the conditions for Fresnel diffraction are satisfied for output field positions nearby the propagation direction of the light. Field of Fresnel diffraction of a point source:

$$U(x', y') = \frac{e^{ikz}}{i\lambda} \frac{\Delta z_1}{\sqrt{\Delta z_1^2 + x'^2 + y'^2}} e^{\frac{ik}{2\Delta z_1}(x'^2 + y'^2)} \tag{1}$$

When comparing the nearby points in x',y', this approximates to:

$$U(x', y') = \frac{e^{ikz}}{i\lambda} e^{\frac{ik}{2\Delta z_1}(x'^2 + y'^2)} \tag{2}$$

A single weight at x'_1, y'_1 is described as:

$$e^{-(\eta(x' - x'_1)^2 + (y' - y'_1)^2)/\sigma_1^2} \tag{3}$$

With these, we can assemble the representation of the field at the output plane using the Fraunhofer diffraction equation:

$$U(x, y) = \int_{-\infty}^{\infty} \int_{-\infty}^{\infty} U(x', y') e^{\frac{-ik}{\Delta z_2}(xx' + yy')} dx' dy' \tag{4}$$

$$U(x,y)=\int_{-\infty}^{\infty}\int_{-\infty}^{\infty}\frac{e^{ikz}}{i\lambda}\frac{\Delta z_1 e^{-(\eta(x'-x_1')^2+(y'-y_1')^2)/\sigma_1^2}}{\sqrt{\Delta z_1^2+x'^2+y'^2}}e^{\frac{ik}{2\Delta z_1}(x'^2+y'^2)}\frac{e^{ikz}}{i\lambda}e^{\frac{ik}{2\Delta z_1}(x'^2+y'^2)}e^{\frac{-ik}{\Delta z_2}(xx'+yy')}dx'dy' \quad (5)$$

The solution of:

$$\int_{-\infty}^{\infty}e^{i(c_1u'^2-c_2u'u)-c_3(u'-u_1')^2}du'=\sqrt{\frac{\pi}{c_3-c_1 i}}e^{\frac{-c_3+c_1 i}{4(c_1^2+c_3^2)}(c_2^2u^2+4ic_2c_3u_1'u-4ic_1c_3u_1'^2)} \quad (6)$$

using the substitutions of $c_1=\frac{k}{2\Delta z_1}$, $c_2=\frac{k}{\Delta z_2}$, $c_{3y}=\frac{1}{\sigma_1^2}$ for the y direction and $c_{3x}=\frac{\eta}{\sigma_1^2}$ for the x direction.

The above solution may be rearranged as:

$$\sqrt{\frac{\pi}{c_3-c_1 i}}e^{-c_3u_1'^2}e^{\frac{ic_3^2u_1'^2}{c_1}}e^{\frac{-c_2^2c_3}{4(c_1^2+c_3^2)}(u-\frac{2c_1u_1'}{c_2})^2}e^{\frac{ic_1c_2^2}{4(c_1^2+c_3^2)}(u+\frac{2c_3^3u_1'}{c_2c_1})^2} \quad (7)$$

or

$$Ae^{i\theta}e^{\frac{(u-u_2)^2}{s_{amp}^2}}e^{\frac{i(u-u_{i2})^2}{s_{phase}^2}} \quad (8)$$

where $s_{amp}=\sqrt{\frac{4(c_1^2+c_3^2)}{c_2^2c_3}}$, $s_{phase}=\sqrt{\frac{4(c_1^2+c_3^2)}{c_1c_2^2}}$, $u_2=\frac{2c_1u_1'}{c_2}$, and $u_{i2}=-\frac{2c_3^3u_1'}{c_2c_1}$

The precursor $Ae^{i\theta}$ represents a static scaling factor for the amplitude and phase offset, and the rest of the expression represents Gaussian amplitude spread s_{amp} centered at u_2 and quadratic phase spread s_{phase} centered at u_{i2}.

After substitution for x, y and rotation, the field incident on the photodiode plane is given by:

$$U(x,y)=\frac{e^{\frac{(x-x_2)^2}{s_{ampx}^2}}e^{\frac{i(x-x_{i2})^2}{s_{phasex}^2}}e^{\frac{(y-y_2)^2}{s_{ampy}^2}}e^{\frac{i(y-y_{i2})^2}{s_{phase}y^2}}e^{ix_1'(x-x_{i2})/\Delta z_1}}{\sqrt{c_{3y}-ic_1}\Delta z_1\sqrt{\eta}(s_{ampx}^2s_{ampy}^2)^{\frac{1}{4}}} \quad (9)$$

where $s_{ampx}=\sqrt{\frac{4(c_1^2+c_{3x}^2)\eta}{c_2^2c_{3x}}}$, $s_{phasex}=\sqrt{\frac{4(c_1^2+c_{3x}^2)}{c_1c_2^2}}$, $x_2=\frac{2c_1x_1'}{c_2}$, $x_{i2}=-\frac{2c_{3x}^3x_1'}{c_2c_1}$, $s_{ampy}=\sqrt{\frac{4(c_1^2+c_{3y}^2)}{c_2^2c_{3y}}}$, $s_{phasey}=\sqrt{\frac{4(c_1^2+c_{3y}^2)}{c_1c_2^2}}$, $y_2=\frac{2c_1y_1'}{c_2}$, and $y_{i2}=-\frac{2c_{3y}^3y_1'}{c_2c_1}$.

In order to minimize crosstalk between adjacent weights, the most important parameters are s_{ampx} and s_{ampy}, which describe the spread of a weight on the PD plane. By choosing parameters such that total spread and crosstalk off-axis is minimized and the total integrated signal on PDs is maximized (SUPP. FIG. 22), we can optimize the performance of our approach.

This equation approximates the spread on the PD plane from a point source going through the optical

layers. In addition to this, the total spread is increased by two additional factors: the change in spread due to LED size and the frequency spread of the wavelength.

The width $\pm\omega_0/2$ of the LED die can be described as a shift of basis where $x_0 \to x_0 \pm \omega_0/2$, which is approximately equal to $x_1' \to x_1' \pm \omega_0/2$. This shift of basis results in:

$x_2 = \frac{2c_1(x_1' \pm \omega_0/2)}{c_2}$

The change in the real center of $2c_1/c_2 = \Delta z_2/\Delta z_1$ is exactly equal to the geometric shift from raytracing and so we approximate these spreads using a convolution of the intensity with a square of size $\omega_0 \Delta z_2/\Delta z_1$. The change in spread due to wavelength:

$\delta k = \frac{2\pi}{15nm}$

causes the real spread of the amplitude s_{amp} to change depending on the λ, Δz_1, and σ_1, with a maximum change in the spread from 1 to $\frac{\delta\lambda}{\lambda}^2$. In the case of our parameters, the value is $\frac{0.35\delta\lambda}{\lambda}^2$ which results in a relatively minor $\pm 2\%$ change in the spread of the output region as a function of the input wavelength.

With these expressions, we now estimate the total crosstalk as a function of the field offset. The field due to an adjacent weight positioned $\delta 1$ away from x_1' or y_1' has the same s_{amp} and s_{phase} but with $x_2 = \frac{2c_1 x_1'}{c_2}$, $x_{i2} = -\frac{2c_3^3 x_1'}{c_2 c_1}$. This calculation suggests, upon the scale-up of our approach, there is a limited range of angles that can be efficiently used for matrix multiplication before diffractive effects dominate and limit useful computation. To surpass these limitations, the use of additional optics for beam-shaping/beam-steering would be required.

Supplementary Note 4: Optical propagation

We simulate the optical layers of our approach using two different optical propagation methods, a Monte Carlo raytracing approach (no diffraction) and using a modified angular spectrum propagation method. Code implementing both these approaches are included in the repository linked in Code Availability.

Monte Carlo Raytracing: Our Monte Carlo Raytracing approach samples individual rays with a starting position and propagation direction from a known distribution for each LED and propagates them through each optical component within our system until they intersect the photodiode plane. The distribution of rays emitted from an LED is modeled using a uniform distribution in space within the LED die of width w_0 and the angular distribution is taken from the WURTH datasheet. These rays then propagate a distance Δ_{z1} until incident with the weight matrix W. The propagation vector is described is $\Delta_{z1}[tan(\theta)sin(\phi), tan(\theta)cos(\phi), 1]$ where θ, ϕ are the heading angle of the ray. The weight matrix is represented as an amplitude mask with a transparency equal to individual weights. A ray passing through this mask is mapped to a corresponding transparency, which represents the probability of the ray being absorbed within this layer. Fresnel equations are used to determine if total internal reflection occurs due to the incident angle of the ray. These rays are further propagated a distance Δ_{z2} until incident with the photodiode plane, where they are binned.

Modified angular spectrum propagation: Simulation of our system using the angular spectrum method models the emission of an incoherent light source as the averaged intensity response of point sources sampled over the die size w_0. We extend this to include the emission of an LED by averaging the output of multiple propagations following the distribution of wavelengths λ given by the LED datasheet.

Angular spectrum propagation was implemented using the Rayleigh-Sommerfeld (RS) diffraction integral with the propagator $\frac{dze^{ik\sqrt{dz^2+\rho^2}}}{i\lambda(dz^2+\rho^2)}$. The sampling constraint for RS propagation is given as $\lambda\sqrt{\frac{\Delta z^2+L^2/2}{L^2}} \approx 0.8\lambda$ where L is the field size. Given these constraints, we tiled the angular spectrum propagation to effectively sample the output region. As before, weight matrices are described using amplitude masks.

Supplementary Note 5: Throughput and Efficiency

The designed system serves as a prototype for demonstrating stackable non-linear layers along with an incoherent optical matrix-vector multiplication. However, the performance of the system can be evaluated and compared to other existing optical and electronic implementations by calculating the throughput and efficiency of the system.

Throughput represents the number of units of information that a system can process in a given unit of time. In the context of neural networks, throughput is measured by the number of operations per second. Each operation refers to a multiply and accumulate (MAC) operation, where the input signal from the previous layer is multiplied with the learned weights of the current layer, and the products for a given weight are summed together. Efficiency, on the other hand, captures the energy used for processing a unit of information per unit time and is represented in operations-per-second-per-watt. It provides a measure of how effectively the system utilizes energy resources.

To calculate these parameters for the system, the following steps were followed:

1. Calculate throughput for the system: Throughput was calculated based on the number of neurons in each layer of the designed optoelectronic neural network and the frequency of operation of the system.
2. Measure the power consumption by the experimental intermediate circuit
3. Calculate the expected power consumption of the scaled-up model
4. Calculate the efficiency: The efficiency of each system was calculated based on the throughput and power consumption of the system for the current experimental setup as well as the sacled-up model

The details for each of these steps are provided in the following sections.

5.1 Throughput Calculation

The implementation presented in this work consists of multiple layers within an optoelectronic neural network. Each layer is subdivided into two components: the electronic and the optical. The electronic component includes a photodetector, amplification electronics, and a light source, while the optical component consists of a mask that implements the weights. Matrix-vector multiplication and accumulation operations occur between the light emitter of one layer and the detector of the subsequent layer. Consequently, the total number of operations in the system is determined by the number of light emitters in the current layer and the number of photodiodes in the following layer.

Consider an intermediate layer where $n \times n$ light emitters project to $m \times m$ photodetectors. By design, the number of output neurons is $\frac{m}{2} \times m$, since two photodiodes are required to implement the rectified linear unit (ReLU) nonlinearity. The total number of operations at any given time step can be expressed as:

$$\text{Throughput} = \frac{f}{2}\left(m^2 n^2 + m^2\right) \tag{10}$$

where the system operates at a frequency of f Hz. The first term corresponds to the optical matrix-vector multiplication and the second term is the electronic integration and ReLU operation. This arrangement is used for the experimental demonstration LED grid size of 8×8 projecting to a photodetector grid size of 8×8, operating at 800kHz. Putting this into $(f/2)(m^2n^2 + m^2)$, we calculate a throughput of 1.7 GOPS.

For the scaled-up model, we have changed the geometry such that the number of output neurons equals the number of input neurons, or $m^2 = 2n^2$. With this constraint, and given that the system is square, we instead have:

$$\text{Throughput} = f \cdot (n^2 + 1)n^2 \tag{11}$$

For the scaled-up simulation with an LED grid size of 32×32 projecting to a photodetector grid size of 48×48 operating at 10MHz, we use the photodiode arrangement shown in SUPP. FIG. 20, resulting in 32×32 output neurons for a throughput of $f(n^2 + 1)n^2 = 10.5$ TOPS.

5.2 Measured Power Drawn by Different Components in the System

Since all power consumption in the optoelectronic neural network system stems from the electronic circuit, estimating the power draw of this circuit is crucial for calculating the system's efficiency. We experimentally measured the power consumption using a circuit implementing one optoelectronic neuron on a matrix breadboard, as shown in SUPP. FIG. 5b, and inserted probes to measure the current through the power terminals of the operational amplifiers and the output branch using a microammeter.

To measure the power consumption accurately, we divided the circuit into four sub-circuits, as illustrated in SUPP. FIG. 27: the photodiode detection stage (PD), two operational amplifier stages (OA1 and OA2), and the output stage (OUT). We calculated the power draw for each sub-circuit individually, then summed these values to obtain the total power consumption. Given that the system comprises of 32 identical circuits, the total power draw of the system can be calculated as:

$$P_{\text{Total}} = 32 \times (P_{\text{PD}} + P_{\text{OA1}} + P_{\text{OA2}} + P_{\text{OUT}}) \tag{12}$$

The power draw for each sub-circuit is tabulated in SUPP. TABLE 6 for an illumination intensity of 200 mW cm^{-2}. The overall power consumption as a function of the input intensity of a calibrated light source is provided in SUPP. FIG. 28.

The power consumption in the photodiode section is primarily due to the dark current in the photodiode, which is given by:

$$P_{\text{PD}} = 2(V_r \cdot I_d) \tag{13}$$

For the SFH 2704 photodiode used in the circuit, where the maximum dark current i_d is 25 nA, the resulting maximum power draw is 125 nW at a reverse bias voltage of 5V. Since this value is negligible compared to

the other power draws in the circuit, it can be safely disregarded in the total power calculation. The power draw in the output branch, which is primarily influenced by the resistive load, can be calculated as:

$$P_{\text{OUT}} = I_{\text{OUT}}^2 \cdot R_{\text{OUT}} \tag{14}$$

where I_{OUT} is the current in the output branch, measured via a microammeter, and R_{OUT} is the resistance of the output branch, which corresponds to R_5 in the circuit shown in SUPP. FIG. 27. The power draw in the two operational amplifier stages was measured experimentally. The steps used for measuring power consumption in these stages is depicted in SUPP. FIG. 29. The power draw through the first operational amplifier, P_{OA1}, was determined by disconnecting the power supply to the second op-amp in the circuit (LM358) and measuring the current at the positive and negative power supply terminals of the operational amplifier. Assuming ideal operational amplifier behavior, where no current flows into the input terminals, the current measured at these terminals is sufficient to calculate the power draw:

$$P_{\text{OA1}} = (I_{\text{OA1+}} + I_{\text{OA1-}}) \cdot V_{\text{OA1}} \tag{15}$$

Here, $I_{\text{OA1+}}$ and $I_{\text{OA1-}}$ are the currents measured at the positive and negative power supply terminals, respectively, and V_{OA1} is the supply voltage to the operational amplifier. After reconnecting the power supply to the second op-amp stage, the current was measured again at the positive and negative terminals, under the same assumption of no current flow into the input terminals. The power draw in the second op-amp stage, P_{OA2}, was then calculated as follows:

$$P_{\text{OA2}} = (I_{\text{OA2+}} + I_{\text{OA2-}}) \cdot V_{\text{OA2}} - P_{\text{OUT}} - P_{\text{OA1}} \tag{16}$$

In this equation, $I_{\text{OA2+}}$ and $I_{\text{OA2-}}$ represent the currents measured at the positive and negative power supply terminals, respectively, and V_{OA2} is the symmetric rail-to-rail supply voltage of the operational amplifier.

Using this fitted model of the total power consumption, we estimated the power draw for a neuronal subcircuit for an incident net light intensity of 10 mW cm^{-2}, corresponding to the typical illumination level in our experimental conditions, to be 4.6mW, which closely matches the measured power draw at these illumination levels (SUPP. TABLE 7). From this value, we obtain:

$$P_{Total} = 32 \times 4.6\text{mW} = 147\text{mW} \tag{17}$$

However, there are additional components in the system whose power consumption has not been included. These components include the spatial light modulator (Holoeye LC2012 [1]), which has a constant power draw of 4W from the wall, regardless of the displayed mask. This energy consumption primarily arises from the display driver since the liquid crystal cell in the spatial light modulator is field-driven. We exclude this power draw from our total calculations because we also demonstrate the use of passive dithered photolithography masks

for implementing weights, as shown in SUPP. FIG. 1. The use of a spatial light modulator for implementing the network is mainly for experimental convenience. Additionally, the input to the network and the output readout involve a digital-to-analog converter (DAC) and an analog-to-digital converter (ADC), respectively. We used dedicated National Instruments DAC and ADC modules for this purpose. The ADC PXIe 6355 has a listed power draw of 1.6W, while the DAC PXIe 6739 draws 3W under operating conditions. We did not include these power draws in our final calculations, as the choice of components was based on availability. However, more energy-efficient DAC and ADC modules are available, and with ongoing developments, it is possible to obtain modules that consume as little as 10μW of power per channel in a lab setting [2].

5.3 Power estimation for an idealized scaled-up model

The estimated power consumption of an intermediate layer in the scaled-up model is the sum of contributions from the LED load as well as from the detection/amplification electronics. We can calculate an estimate of these two power draws independently, keeping in mind that the design for the amplification electronics should be sufficient to drive the output.

First, we determine the minimum optical power necessary for the operation of the system. The minimum optical power is either bandwidth or shot noise limited. We can calculate the total bandwidth of the system from the bandwidth of the two stages used in the cascaded amplifier circuit (SUPP. FIG. 30). The electronic bandwidth of the circuit, f_{total}, can be determined from the individual bandwidths of the transimpedance stage, f_{TIA}, and the transconductance stage, f_{TCA}, using the equation:

$$f_{\text{total}} = \sqrt{f_{\text{TIA}}^2 + f_{\text{TCA}}^2} \tag{18}$$

Given the circuit's total bandwidth, f_{total}, the minimum optical power detectable by the SFH2704 photodiode can be estimated using the noise-equivalent power (NEP) provided in the photodiode's datasheet:

$$P_{\text{min}} = \text{NEP} \cdot \sqrt{f_{\text{total}}} \tag{19}$$

As this minimum detectable power is on the order of tens of picowatts (SUPP. FIG. 24c) and is several orders of magnitude lower than the shot noise limits (nW), the electronic bandwidth constraint does not limit the minimum detected optical power in our system and is rather dictated by the shot noise of the detector. The load power is determined by the minimum optical power needed to overcome shot noise. The photocurrent I_p detected in the scaled-up model photodiode at each layer with 8-bit precision is given by:

$$I_{ph} = 255^2 \cdot hf \cdot F \cdot R = (65025)(3.82\ e^{-19}\text{J})(10\ \text{MHz})(0.34\ \text{A W}^{-1}) = 84\ \text{nA} \tag{20}$$

where hf is the photon energy, F is the operation frequency, and R is the photodiode spectral sensitivity. In comparison, the dark current is 0.1 nA, contributing additional noise of approximately 0.3 bits. This corresponds to a light intensity on the photodiode of $I = I_{ph} \cdot \text{A} \cdot \text{R}^{-1} = 166\ \text{nW mm}^{-2}$, where $A = 1.5\text{mm}^2$ is

the photodiode area. To achieve a light intensity of 166nW mm^{-2} at the photodiode plane, we integrate the expected signal from all $32 \times 32 = 1024$ LEDs to the photodiodes, with an average collection efficiency C for a given LED-PD pair of 2.8 e^{-5}. Using these values and a normalized neuronal weight value of $w_0 \approx 0.1$, we estimate the per LED power consumption:

$$P_l = \frac{I \cdot A}{w_0 \cdot C \cdot n_{LED} \cdot E_l} = \frac{(166\text{nWmm}^{-2})(1.5\text{ mm}^2)}{(0.1)(2.8\ e^{-5}))(1024)(0.541)} = 160\mu\text{W} \tag{21}$$

Where E_l is the best wall-plug efficiency for an LED [3, 4]. Multiplying by the number of LEDs gives a total load power consumption of 163mW.

Second, the power consumption of the detection/amplifier circuits in the scaled-up model is the sum of the contributions from the transimpedance amplification stage and the transconductance amplification stage. As described earlier, we have proposed, designed, and demonstrated the operation of such a faster circuit using off-the-shelf components by means of a SPICE simulation (SUPP. FIG. 23). However, application-specific integrated circuits (ASICs) that implement these operations will be essential for minimizing power consumption in an experimental realization of the scaled-up model, as discrete operational amplifiers have a significant quiescent power draw that increases linearly with frequency in a log-log plot [5]. A proposed schematic for such an ASIC implementation is shown in SUPP. FIG. 30. These circuits need to meet the requirements for the circuit that have been tabulated (SUPP. TABLE 8). We found suitable integrated circuit transimpedance and transconductance amplifier implementations that match the requirements of this design.

The transimpedance amplifier implementation [6] was designed for a low-power wearable optoelectronic biomedical sensor application with input from a silicon photodiode. This implementation was designed to operate at 1.15 MHz with a gain of 124 dBΩ. We can interpolate the available gain from the design when operating at a higher frequency from the frequency versus magnitude of gain data (Figure 4 in [6]). We obtain a gain of 90 dBΩ for the circuit operating at 10 MHz. The output from two such identical transimpedance amplifiers is connected to a transconductance stage for voltage to current conversion.

The transconductance amplifier implementation [7] was designed as a differential input buffered and external transconductance amplifier (DBeTA) for low power biomedical applications. This design makes it suitable for the transconductance stage as this inherently implements subtraction and voltage-to-current conversion with unity gain. This design is capable of driving the required output, which in this case is the current through the output LED. The power required for driving the LEDs in the circuit was calculated above to be 160 μW. For a supply voltage of 3.5 V, the current needed to drive the LED ranges from $0 - 46$ μA, which is within the transconductance maximum current range of 60 μA.

The power draw under the required conditions for the two transimpedance amplifier and the transconductance amplifier is calculated:

$$P_a = 2P_{ti} + P_{tc} = 2(36\mu\text{W}) + 62\mu\text{W} = 134\mu\text{W} \tag{22}$$

where $2P_{ti}$ is the power consumption of a pair of custom-designed transimpedance amplifiers at 90dB gain and P_{tc} is the power consumption for a transconductance amplifier with unity gain. Over all 1024 neurons, the amplifier power consumption is estimated as 137mW. Together the total estimated power for an optoelectronic layer in the scaled-up model is:

$$P_{tot} = 163\text{mW} + 137\text{mW} = 300\text{mW} \tag{23}$$

5.4 Efficiency Calculation

The efficiency of the system is derived from the throughput and power draw calculations as follows:

$$\text{Efficiency (OPS/W)} = \frac{\text{Throughput (OPS)}}{\text{Power Draw (W)}} \tag{24}$$

From the experimental setup demonstrating the optoelectronic neural network operateing at 800kHz with an 8×8 LED array projecting onto a 8×8 photodiode grid we obtain a throughput of 1.7GOPS and a total power consumption of 147mW, we obtain:

$$\text{Efficiency (expt)} = \frac{1.7\text{GOPS}}{0.147\text{W}} = 11.45\text{GOPS/W} \tag{25}$$

Similarly, for the scaled-up model with an LED grid size of 32×32 projecting to a photodetector grid size of 48×48 operating at 10MHz and a total power consumption of 300mW, we obtain:

$$\text{Efficiency (scaled-up)} = \frac{10.5\text{TOPS}}{0.300\text{W}} = 35\text{TOPS/W} \tag{26}$$

Supplementary Tables and Figures

Label	Correlation	Exp. Acc.	Sim. Acc.	Acc. Ratio
0	0.987	0.959	0.959	1.000
1	0.987	0.966	0.974	0.991
2	0.984	0.869	0.859	1.012
3	0.993	0.892	0.882	1.012
4	0.981	0.943	0.933	1.010
5	0.977	0.880	0.880	1.000
6	0.987	0.926	0.926	1.000
7	0.977	0.915	0.932	0.982
8	0.985	0.897	0.897	1.000
9	0.978	0.850	0.860	0.988

Supplementary Table 1: MNIST output correlations for each of the 10 output classes in the full multilayer experiment. The experimental accuracy is compared to the simulated accuracy for the test dataset. The overall accuracy of the simulation test set is 91.2% and the overall accuracy of the experimental test set is 91.1%.

Factor	Symbol	Parameters	Value
Input Current	i_{inp}		
Absolute Luminous Intensity	I_{abs}		1400 mcd
Emission Angle	Ω_{em}		4π rad
LED directional emission	I_γ^{rel}		Extracted from datasheet
Luminous Intensity	I_γ	Input Current (i_{inp})<br>LED directional emission (I_γ^{rel})<br>Angles (α, β)	Extracted from datasheet
Luminous flux generated	Φ_γ	Emission Solid Angle (Ω_{em})<br>Luminous Intensity (I_γ)	$\Phi_\gamma = \Omega_{em} \cdot I_\gamma(i_{\text{inp}}, \Omega)$
Diameter of the lens	d_{lens}		40 mm
Focal Length of the lens	f_{lens}		100 mm
Angle selected by the lens	α_{lens}	Diameter of the lens (d_{lens})<br>Focal Length of the lens (f_{lens})	$\alpha_{\text{lens}} = 2 \cdot \tan^{-1} \frac{0.5 d_{\text{lens}}}{f_{\text{lens}}} = 11.42°$
Solid Angle selected by the lens	Ω_{lens}	Angle selected by the lens (α_{lens})	$\Omega_{\text{lens}} = 2\pi(1 - \cos(0.5 \cdot \alpha_{\text{lens}})) = 0.03$ Sr
Percent flux selected	p_{sel}	Emission Angle (Ω_{em})<br>Angle selected by the lens (Ω_{lens})	$\Phi_\gamma \cdot \frac{\Omega_{\text{lens}}}{\Omega_{\text{em}}} = 0.0287$
Spot area formed on photodiode	a_{spot}		9 mm^2
Radiant sensitive area on the photodiode	a_{pd}		1.51 mm^2
Percent overlap on photodiode	p_{pd}	Spot area formed on photodiode (a_{spot})<br>Radiant sensitive area on the photodiode (a_{pd})	$p_{\text{pd}} = \frac{a_{\text{pd}}}{a_{\text{spot}}}$
Photodiode directional sensitivity	$S_{\alpha,\beta}$	Angle of the incoming rays (α, β)	Extracted from datasheet
Light collected by photodiode	L_{pd}	Photodiode directional sensitivity ($S_{\alpha,\beta}$)<br>Percent overlap on photodiode (p_{pd})<br>Percent flux selected (p_{sel})<br>Luminous Flux (Φ_γ)	$L_{\text{pd}}(\Phi_\gamma \cdot p_{\text{sel}} \cdot p_{\text{pd}} \cdot S_{\alpha,\beta})$
Optical power on photodiode	P_{pd}	Maximum Sensitivity ($K_m = 683$ lm W^{-1} at 555nm)<br>Photopic spectral luminous efficiency curve $V(\lambda)$ which is 1 at 555nm	$P_{\text{pd}} = \frac{L_{\text{pd}}}{K_m \cdot V(\lambda)} = 1.46 \cdot 10^{-3} L_{\text{pd}}$
Spectral sensitivity	S_λ		0.34 $\frac{A}{W}$
Photocurrent generated	i_{pd}	Optical power on photodiode (P_{pd})<br>Spectral sensitivity (S_λ)	$i_{\text{pd}} = P_{\text{pd}} \cdot S_\lambda$

Supplementary Table 2: Factors that play a role in calculating the amount of light incident on the photodiode in the circuit used to calculate the computational efficiency.

**Parameter**	**Symbol**	**Value**
LED die width	w_{LED}	$150\mu m$
LED spacing	s_{LED}	$2.5mm$
Width of optical weight	w_{amp}	$160\mu m$
Weight separation	s_{amp}	$200\mu m$
Photodiode separation	s_{PD}	$2.5mm$
Distance between LED and weight mask	d_1	$3mm$
Distance between weight mask and PD plane	d_2	$34.5mm$
PD width	w_{PD}	$0.5mm$
System magnification	M	$(d_1 + d_2)/d_1$

Supplementary Table 3: Optical parameters used in experimental implementation

**Component**	**Part Number**	**Quantity**
Operational Amplifier	MCP6V66T-E/OT	1
	LM358	1
MOSFET	BSS138PW	1
Photodiode	SFH2704	2
Light Emitting Diode	150040GS73220	1
Resistors	12.5 Ω	1
	10 kΩ	3
	330 kΩ	1

Supplementary Table 4: Component list for implementing one of the 32 independent and identical circuits that make up our printed circuit board that implements the optical neural network.

Parameter	Symbol	Value
LED die width	w_0	$10\mu m$
Width $(1/e)$ of optical weight	σ_1	$25\mu m$
Weight separation	δ_1	$75\mu m$
PD separation	δ_2	$2.5mm$
Distance from LED plane to weight plane	d_1	$2.5mm$
Distance between weight plane and PD plane	d_2	$84.2mm$
Planes at LED, weight mask, PDs	$(x'', y''), (x', y'), (x, y)$	
LED position	$x''_0 = 0, y''_0 = 0$	
Weight position (relative to LED position)	x'_1, y'_1	
PD position (relative to LED position)	$x_2, y_2 = Mx'_1, My'_1$	
PD width	σ_2	$1.2mm$
Wavelength	λ	$500 - 540nm$
Wavenumber	$k = 2\pi/\lambda$	
Distance scaling of off-axis location	η	$1 + \frac{x_1'^2 + y_1'^2}{d_1^2}$
Distance between LED and weight pair	Δz_1	$d_1\sqrt{\eta}$
Distance between weight and PD pair	Δz_2	$d_2\sqrt{\eta}$

Supplementary Table 5: Optical parameters used for scaled-up model.

Stage	Power Draw (mW)
P_{PD}	$400 \cdot 10^{-9}$
P_{OA1}	0.2653
P_{OA2}	7.0853
P_{OUT}	1.6

Supplementary Table 6: Compilation of power drawn by the different stages in the circuit that make up the opto-electronic neural network for an illumination intensity of 200 mW cm^{-2}.

Net optical input (mW)	Input Intensity(mW/cm^2)	Circuit Power(mW)
0.59	39	5.3
0.26	17	4.9
0.14	9	4.6
0.03	2	4.4
0.00	0	4.1

Supplementary Table 7: Compilation of power drawn in a neuronal circuit equivalent to experimental setup at typical experimental illumination levels.

**Property**	**Requirement**	**TIA [6]**	**TCA [7]**	**Combined**
Operating Frequency	10 MHz	10 MHz	10.88 MHz	10 MHz
Supply Voltage	No limitation	1.8 V	±400 mV	
Amplification	73 dB	90dBΩ	1 Ω^{-1}	90 dB
Power Consumption		36 μW	62 μW	134 μW

Supplementary Table 8: Comparison of the required properties for an idealized scaled-up model using integrated circuits with available literature implementations. The two components that make up the circuit are two trans-impedance amplifiers (TIA) and a trans-conductance amplifier (TCA) whose specifications are summarized here.

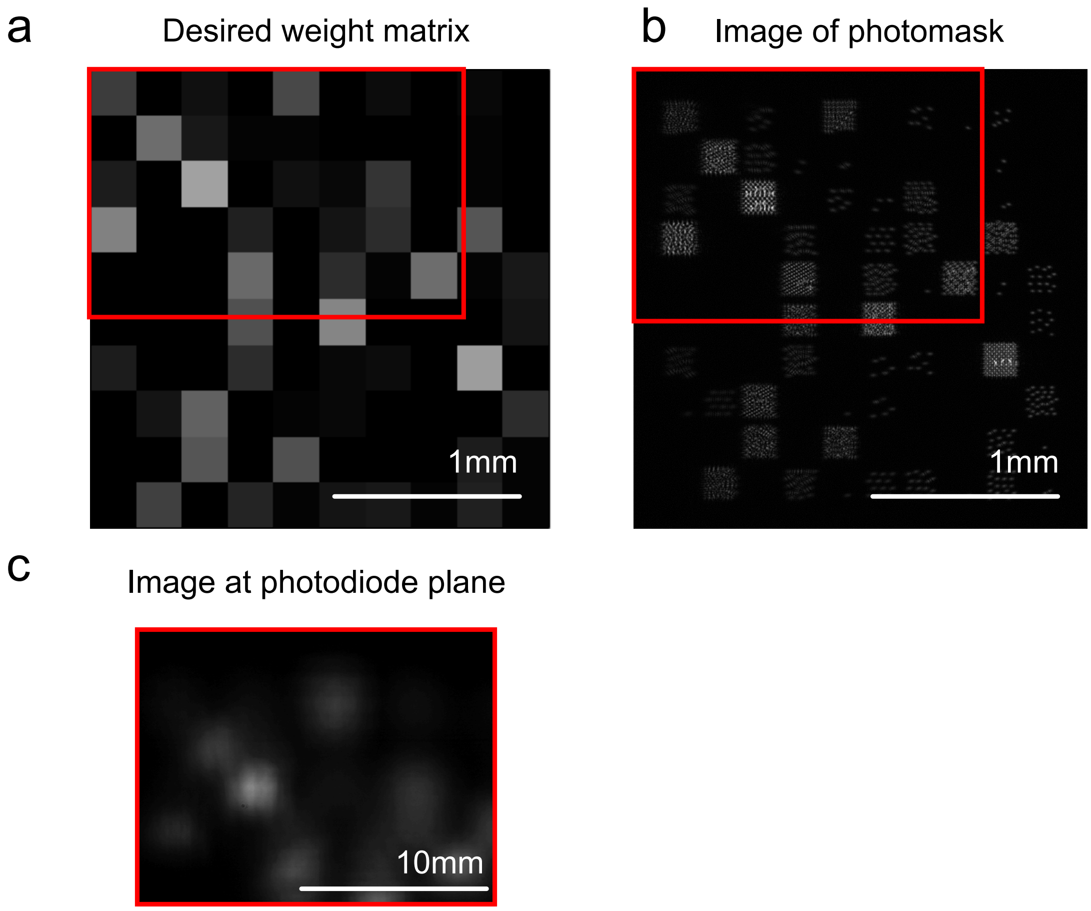

Supplementary Figure 1: Implementation of a passive amplitude mask using binary dithered weights. (a) Desired weight matrix on amplitude mask. (b) High-resolution image of photomask implementing weight matrix with binary dithered weights. (c) Propagated image at output plane showing smoothing of dithered weights.

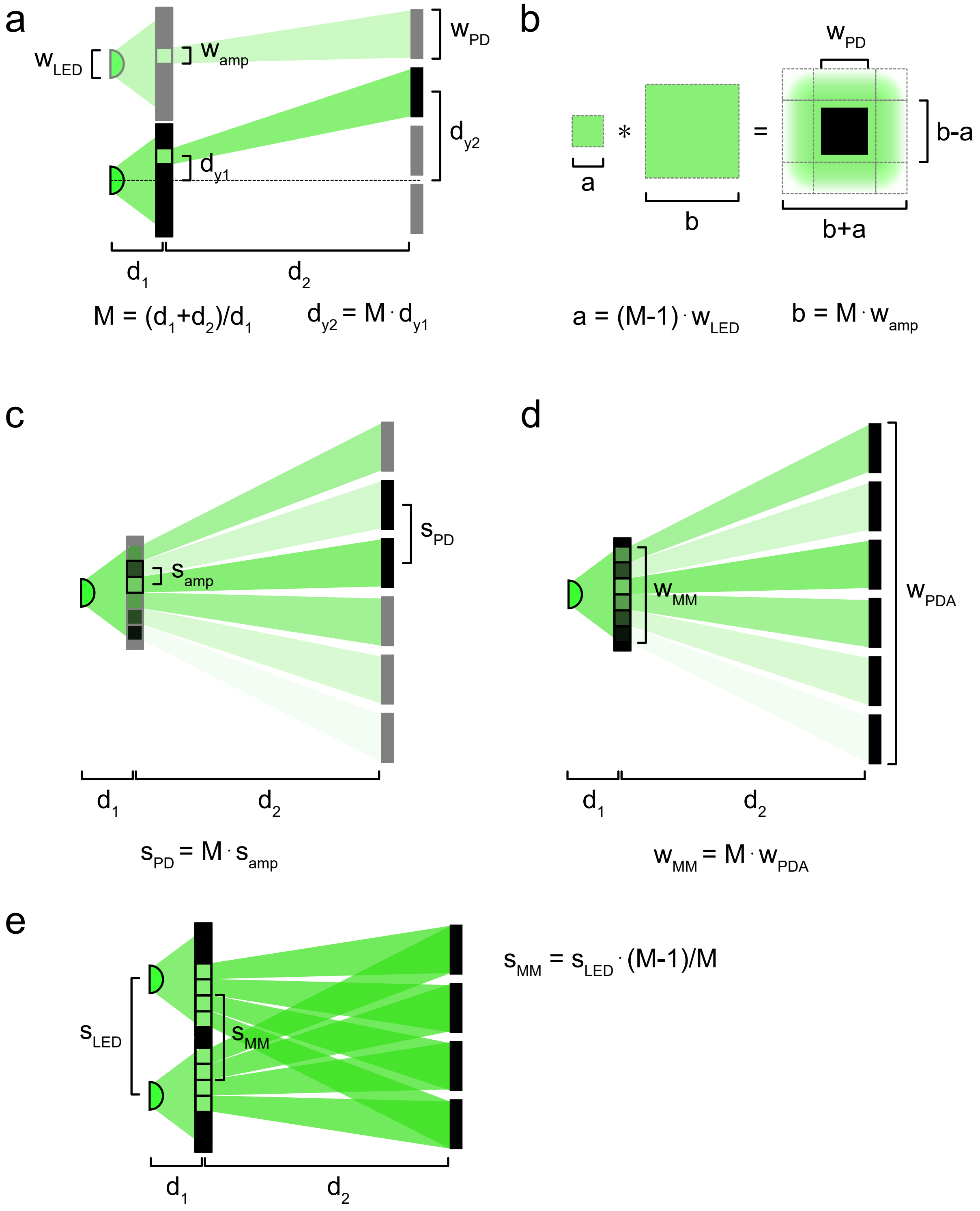

Supplementary Figure 2: Ray-tracing illustrates how a fully-connected optical MVM operation is implemented in our system. (a) Each LED is associated with a subarray on the amplitude mask. The magnification factor M is equal to the relative total propagation distance to the distance between the LED and amplitude mask. (b) The spot size at the detector plane depends on the LED die size, weight size, and magnification factor (not including diffraction). (c) The spacing between photodiodes is equal to M times the spacing between optical weights. (d) The total output region is equal to M times the subarray size. (e) The spacing between subarrays depends on the LED spacing and M.

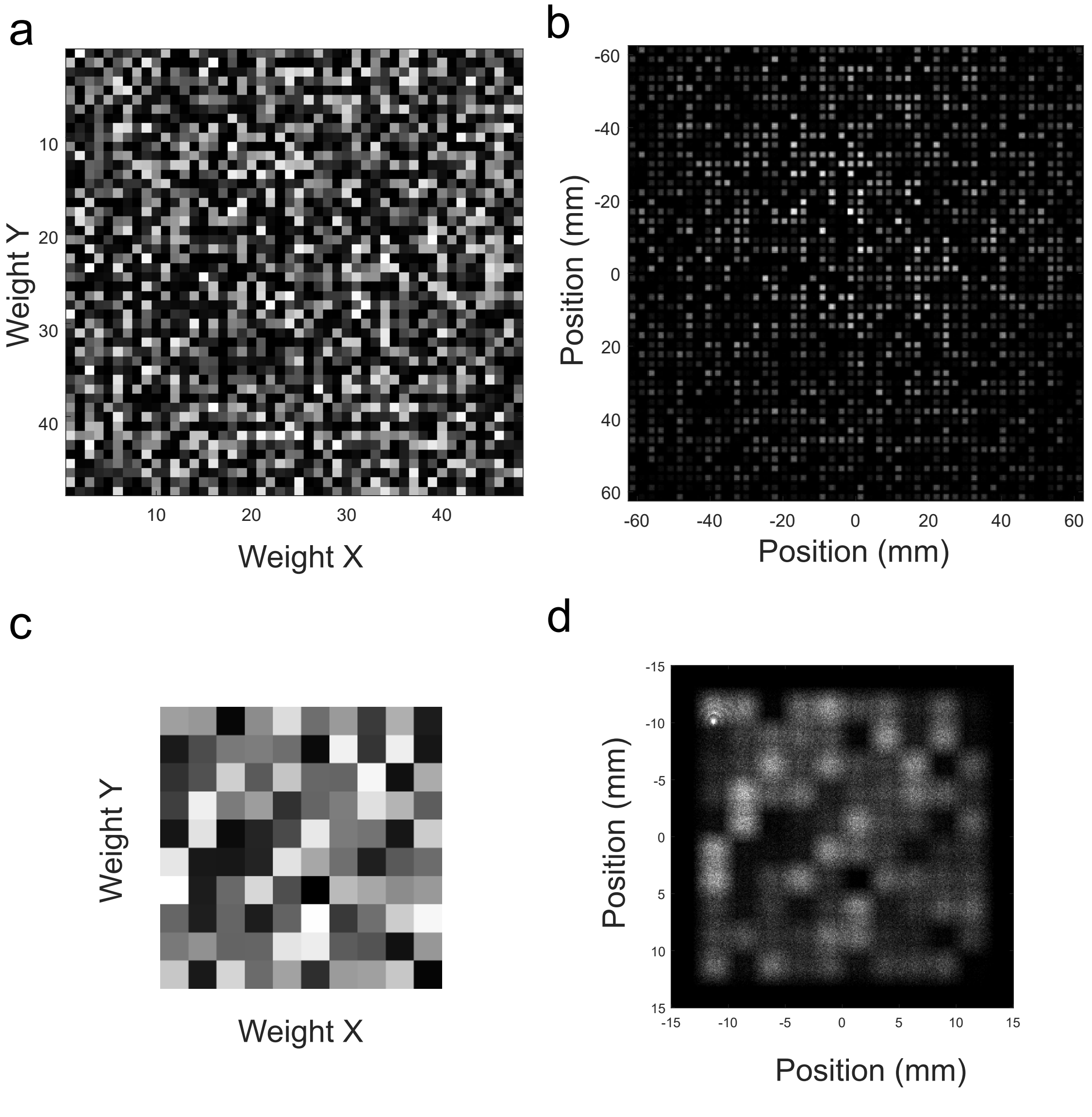

Supplementary Figure 3: Raytracing simulation predictions for two sets of optical parameters. (a) Image of 48×48 optical amplitude weights to be propagated. (b) Raytracing output for weights in (a) with $2.5mm$ PD spacing and $10\mu m$ LED die size. (c) Image of 8×8 optical amplitude weights to be propagated. (d) Raytracing output for weights in (c) with $2.5mm$ PD spacing and $200\mu m$ LED die size.

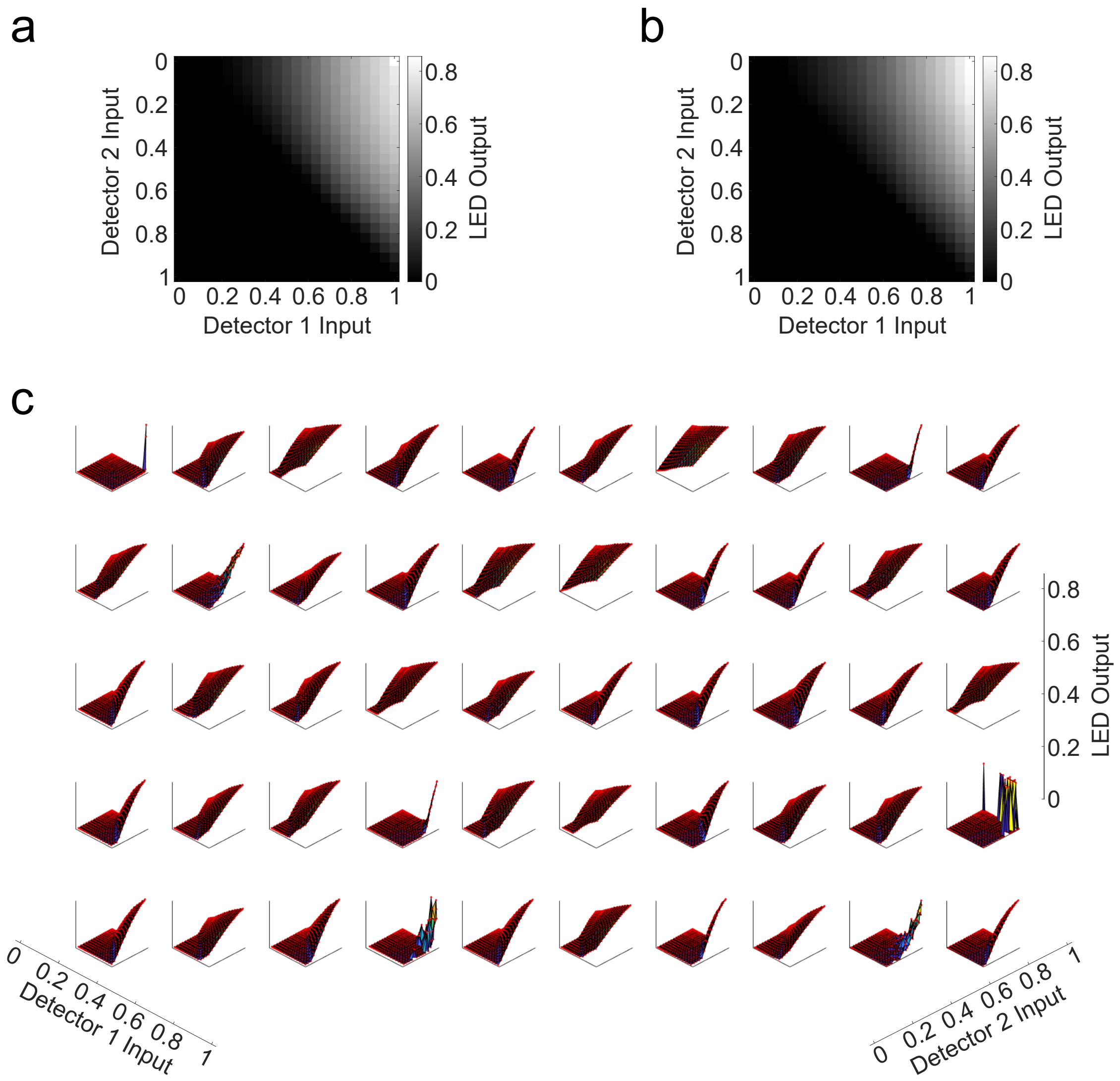

Supplementary Figure 4: Difference $ReLU$ response in circuit to varying photodiode input signals. (a) Experimental (b) Fit of response from (a) to $LED_{output} = ReLU(c_1 I_1 - c_2 I_2 + c3)$. (c) Measured responses of all 50 neurons present on one intermediate board.

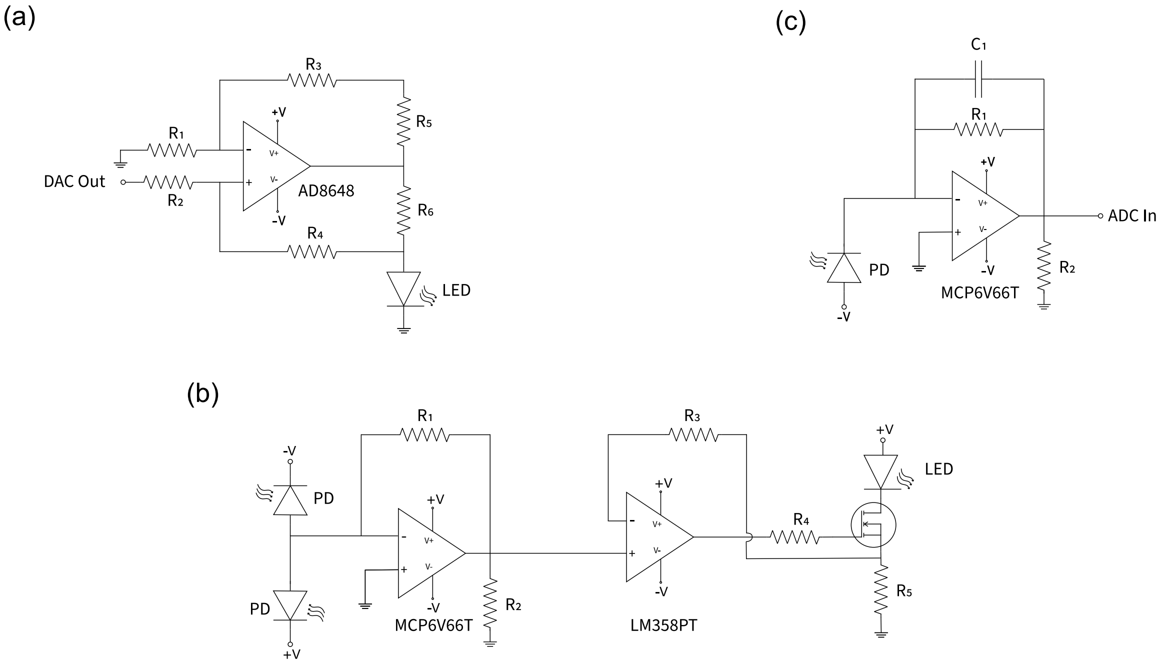

Supplementary Figure 5: Op-amp circuit diagrams used on electronic boards. (a) Driving circuit for data read-in. (b) Read-out circuit for output board. (c) Detection, differencing, and amplification circuit used to drive LEDs in intermediate boards.

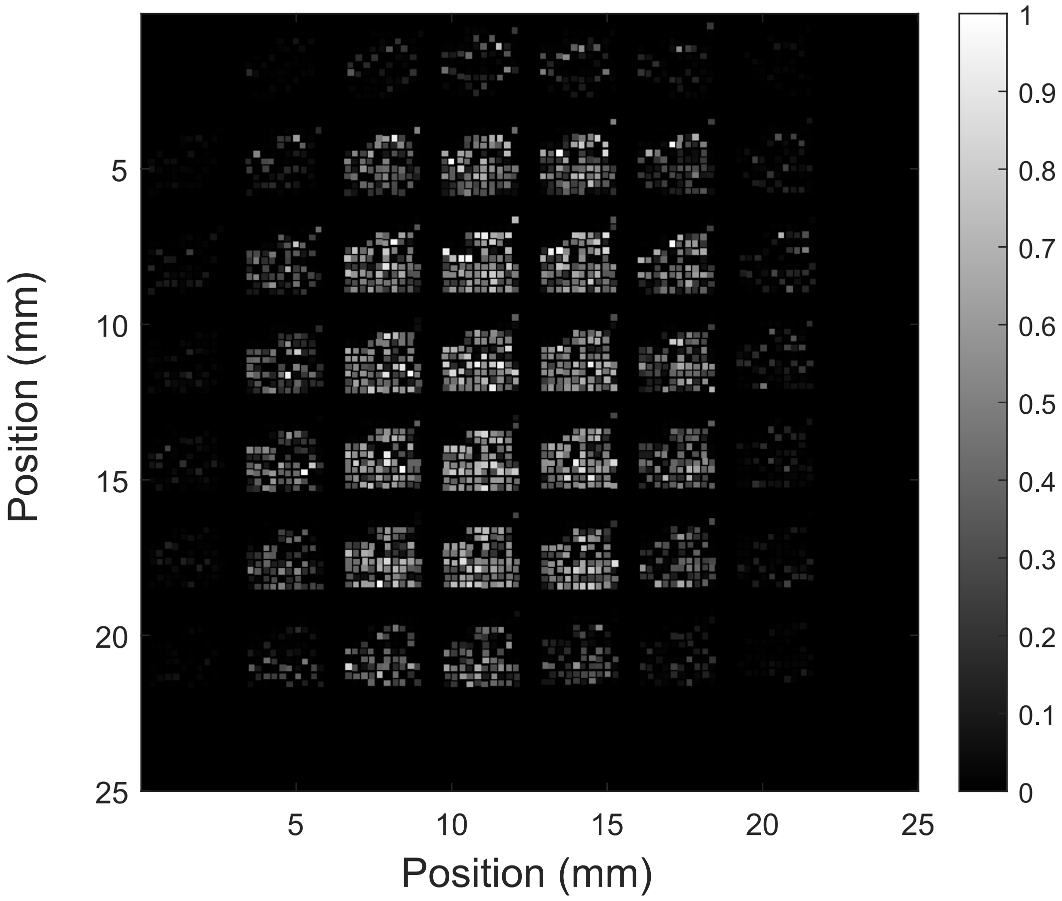

Supplementary Figure 6: Example amplitude mask encoding weights for a network trained on the MNIST digit classification dataset. Individual weights have been shifted to account for exact LED and PD positions.

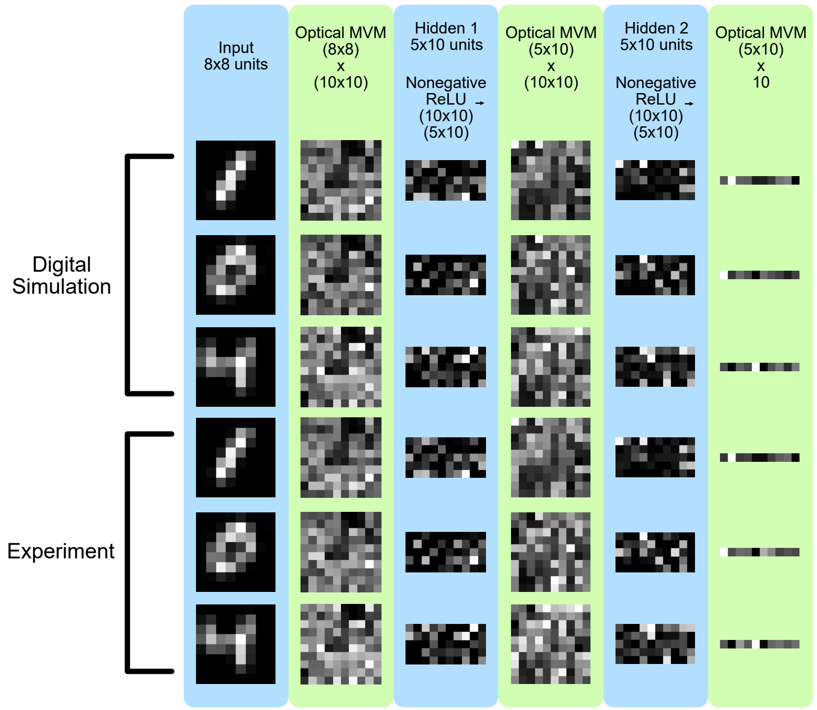

Supplementary Figure 7: Additional propagation examples as described in FIG. 3a.

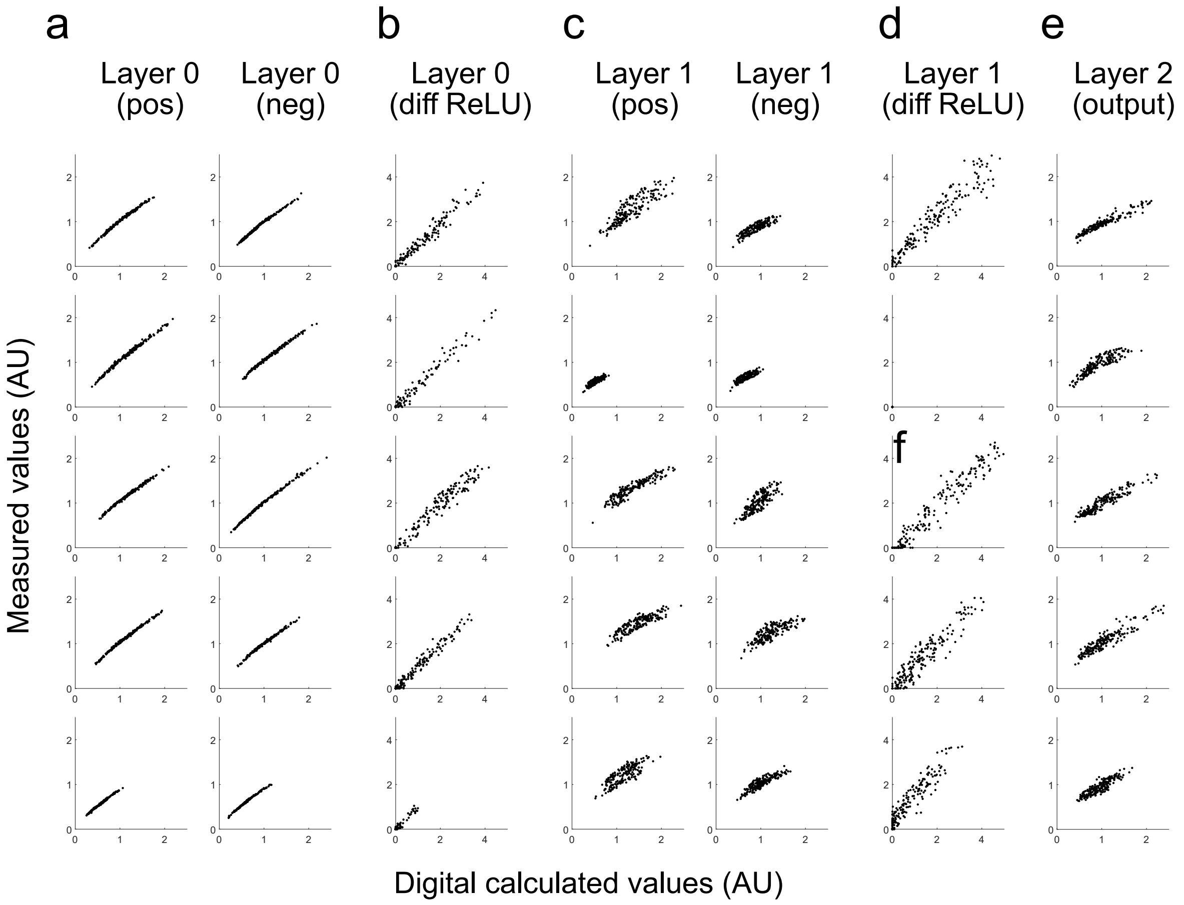

Supplementary Figure 8: Example scatterplots of miniaturized MNIST network neuron activations. Normalized experimental activation versus digital calculated activations are provided after the (a) first optical MVM (b) first differential ReLU (c) second optical MVM (d) second differential ReLU and (e) third optical MVM. The corresponding relative standard deviation of errors to standard deviation of calculated neuron activations are: (a) 0.048, (b) 0.152, (c) 0.145, (d) 0.191, and (e) 0.154.

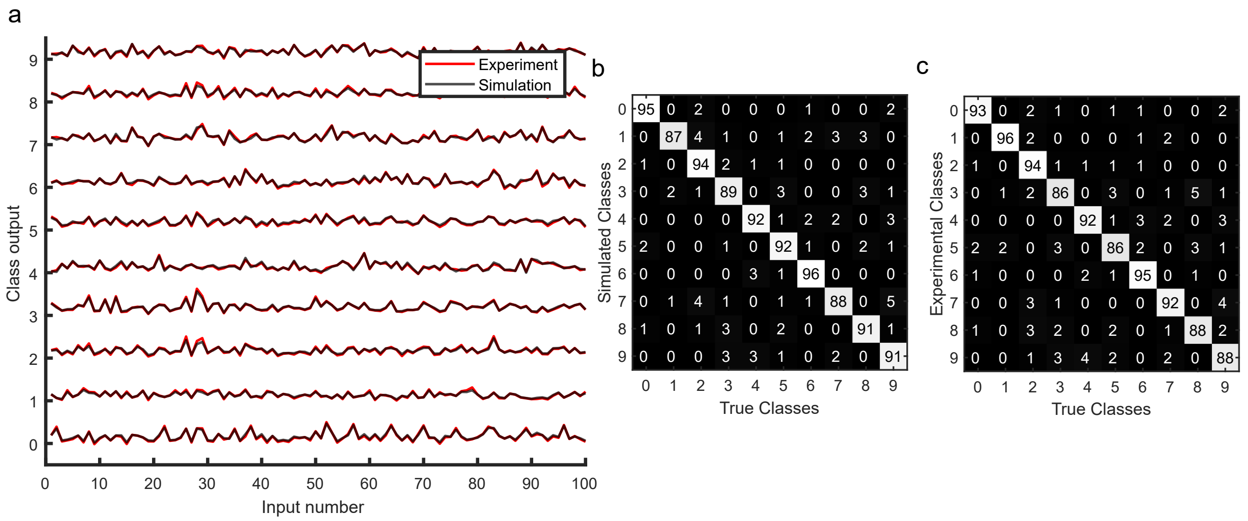

Supplementary Figure 9: Full multilayer optoelectronic neural network output on MNIST classification problem. A fully-connected three layer neural network was trained *in silico* with allowed maximum weights set to experimentally measured values. (a) Comparison between simulation and experiment of the trained network output layer values. (b) Confusion matrix of estimated classes for simulated results in percent. (c) Same as (b), but for experimental results. The overall accuracy of the simulation test set is 91.2% and the overall accuracy of the experimental test set is 91.1%.

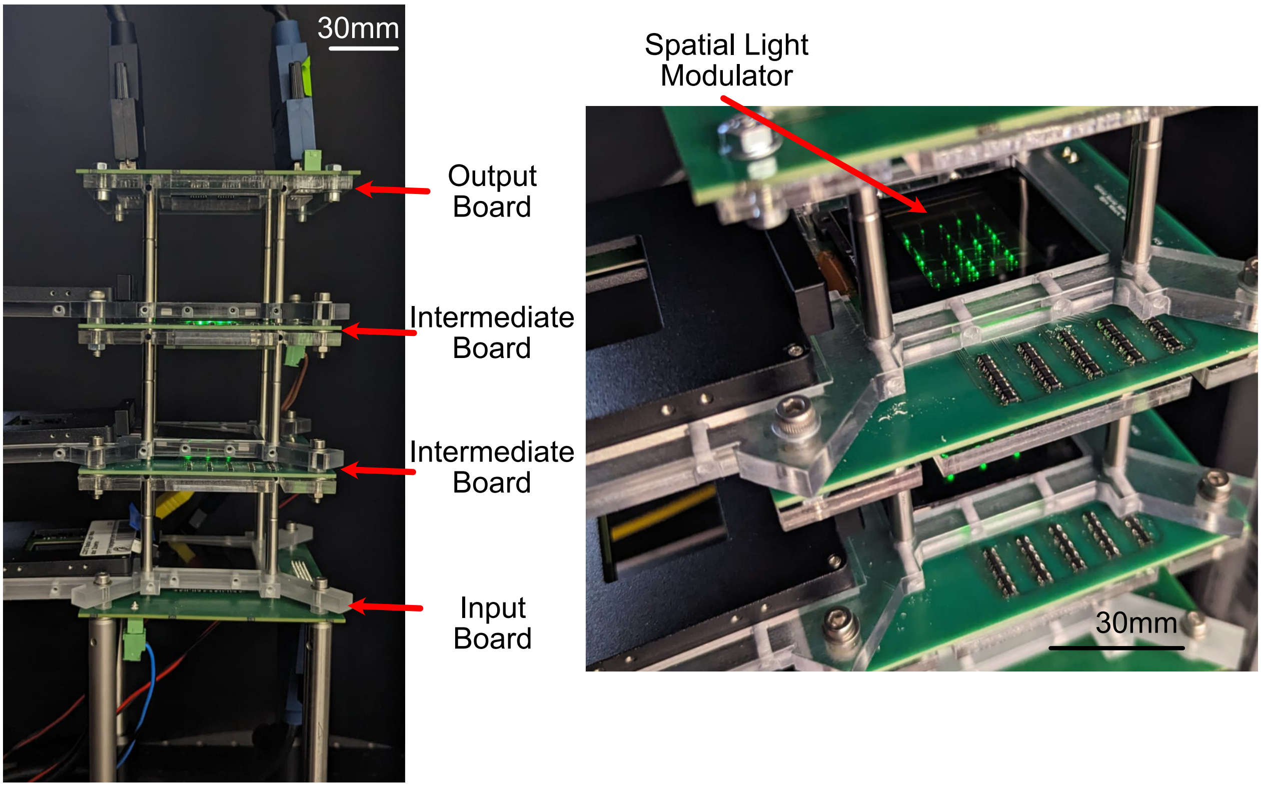

Supplementary Figure 10: Image of experimental setup. Network propagation starts from the input board and propagates upwards through two intermediate boards onto the output board. A transmissive spatial light modulator and a pair of polarizers are used to dynamically encode the amplitude weights.

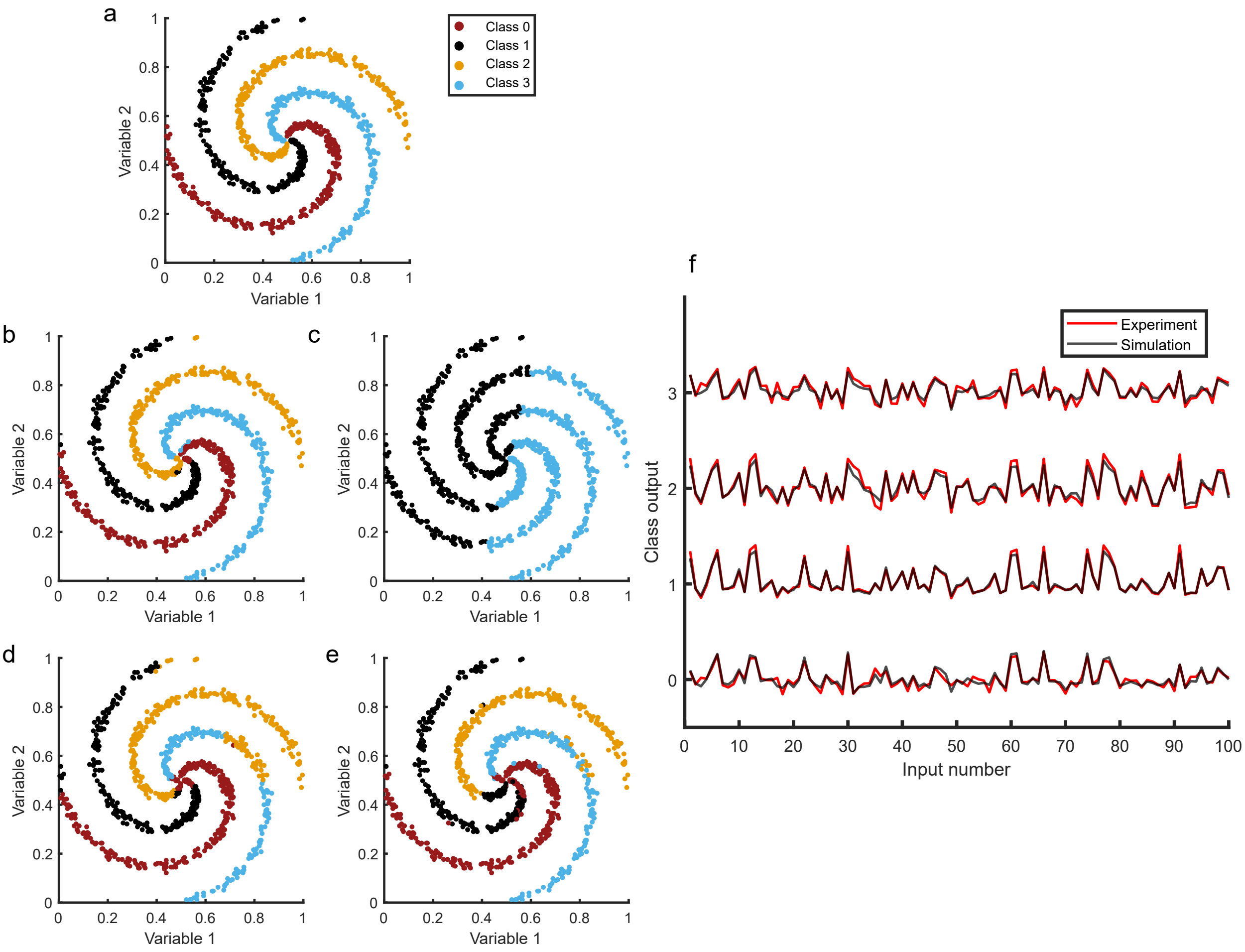

Supplementary Figure 11: (a) A nonlinear four-class spiral data classification problem with two input variables. Each of the classes corresponds to one arm of the spiral. (b) Trained nonlinear network using experimental network parameters as described in FIG. 3a performs with a 96.1% accuracy. (c) The best linear classifier classifies this problem with an accuracy of 30.1%. (d) Simulated network performance using discretized copies of weights from (b) yields a classification accuracy of 87.8%. (e) Experimental classification using the multilayer network obtains a classification accuracy of 86.0%. (f) Comparison between simulation and experiment of the trained network output values.

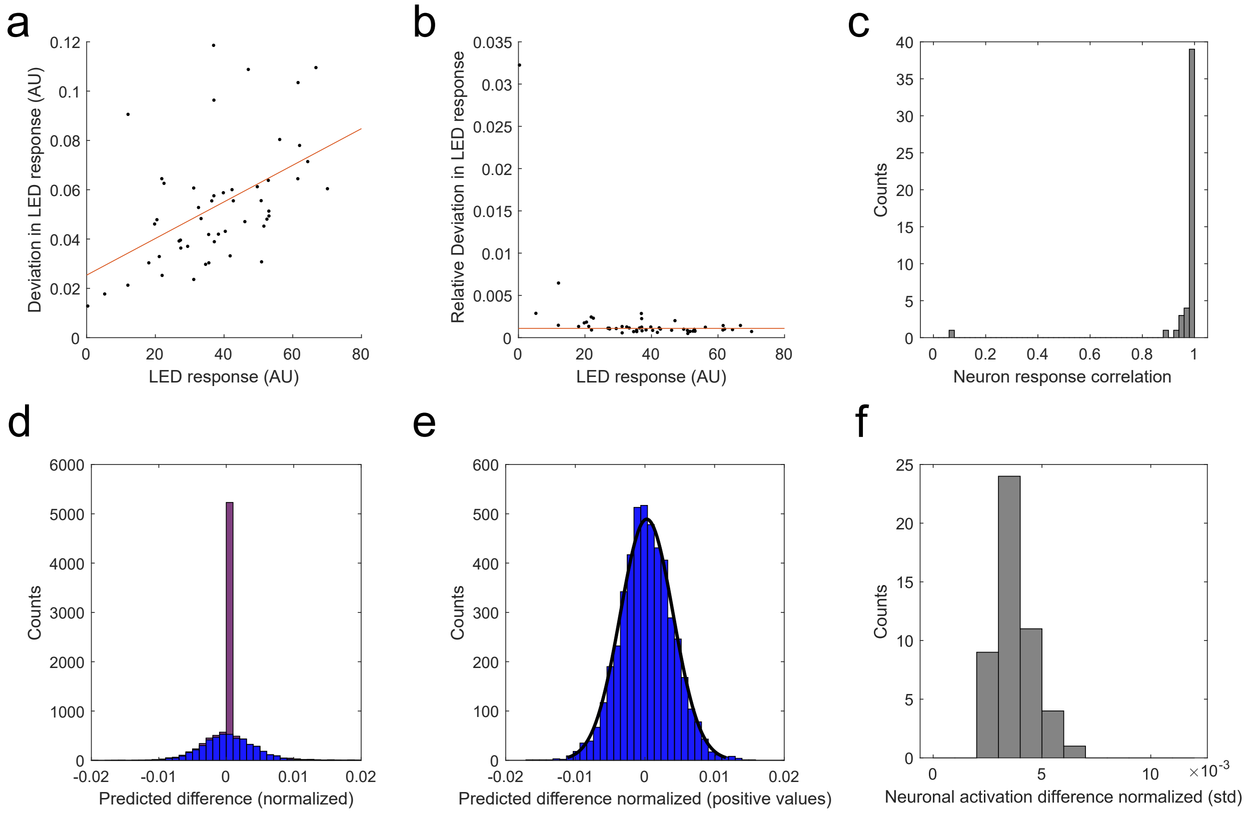

Supplementary Figure 12: Variation and errors in LED response through the first intermediate layer. (a) Scatterplot of the standard deviation in LED output brightness plotted against average LED response for individual neurons. Red line indicates a linear fit of the error response ($y = 0.0007x + 0.025$, $r = 0.49$) (b) Data from (a) normalized by average LED response. Red line indicates median relative deviation (0.0011). (c) Histogram of correlations of predicted values versus LED responses to a set of randomized inputs and weights from the source layer. The predicted responses are calculated from the best fit using a linear weighting and difference ReLU model. (d) Histogram of differences between model estimate and LED responses in (c) normalized by max neuronal response. The distribution is separated into blue (positive model responses) and purple (zero model responses). (e) Blue distribution in (d) fit by a Gaussian (black, $\sigma = 0.0038$) (f) Histogram of standard deviation of percent error between model estimate and LED responses to randomized inputs and weights, as calculated in (e), but for individual neuronal responses.

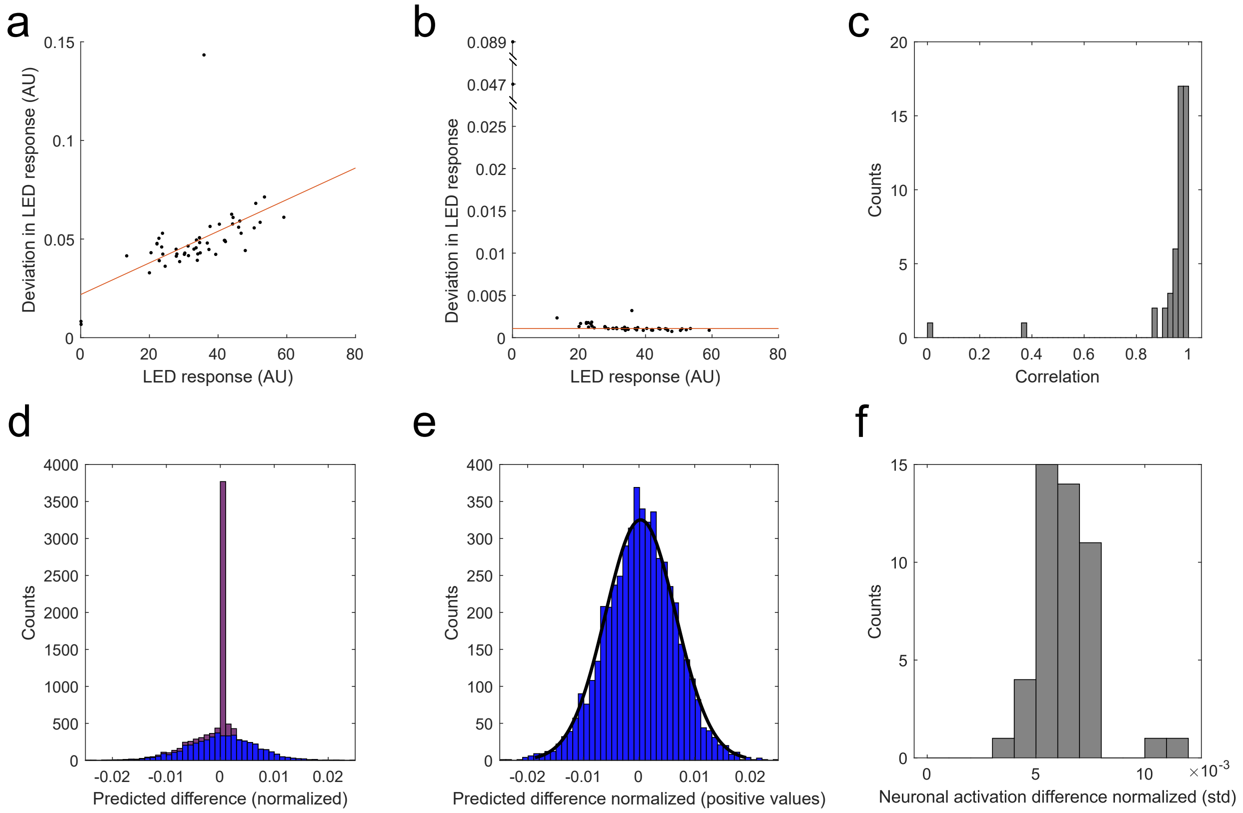

Supplementary Figure 13: Variation and errors in LED response through the second intermediate layer. (a) Scatterplot of the standard deviation in LED output brightness plotted against average LED response for individual neurons. Red line indicates a linear fit of the error response ($y = 0.0008x + 0.022$, $r = 0.55$) (b) Data from (a) normalized by average LED response. Red line indicates median relative deviation (0.0013). (c) Histogram of correlations of predicted values versus LED responses to a set of randomized inputs and weights from the first intermediate layer. The predicted responses are calculated from the best fit using a linear weighting and difference ReLU model. (d) Histogram of differences between model estimate and LED responses in (c) normalized by max neuronal response. The distribution is separated into blue (positive model responses) and purple (zero model responses). (e) Blue distribution in (d) fit by a Gaussian (black, $\sigma = 0.0063$) (f) Histogram of standard deviation of percent error between model estimate and LED responses to randomized inputs and weights, as calculated in (e), but for individual neuronal responses.

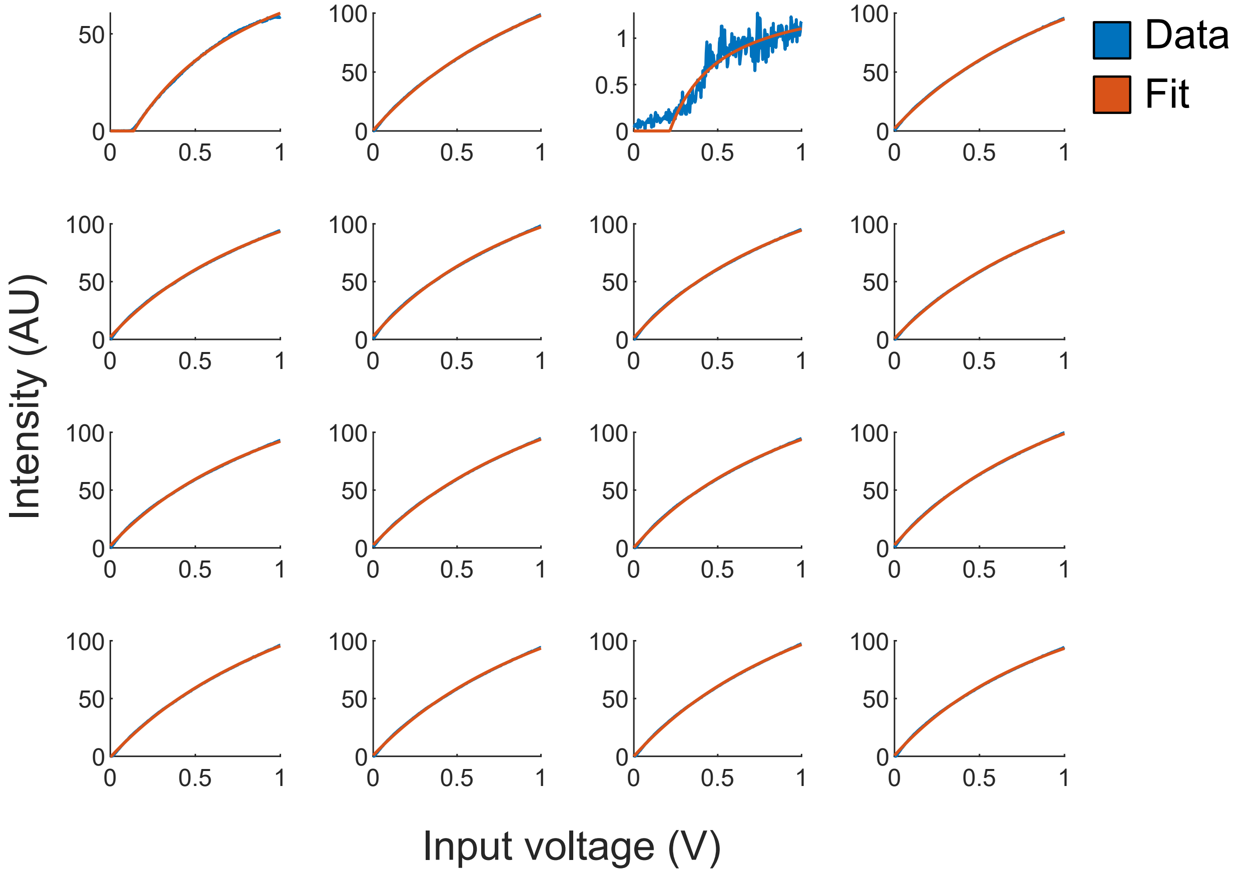

Supplementary Figure 14: Measured responses of sample LEDs from the input board to an input voltage. A example of a malfunctioning unit is seen in row 1, column 3. As individual units are independent, the operation of one malfunctioning unit has no affect on other units.

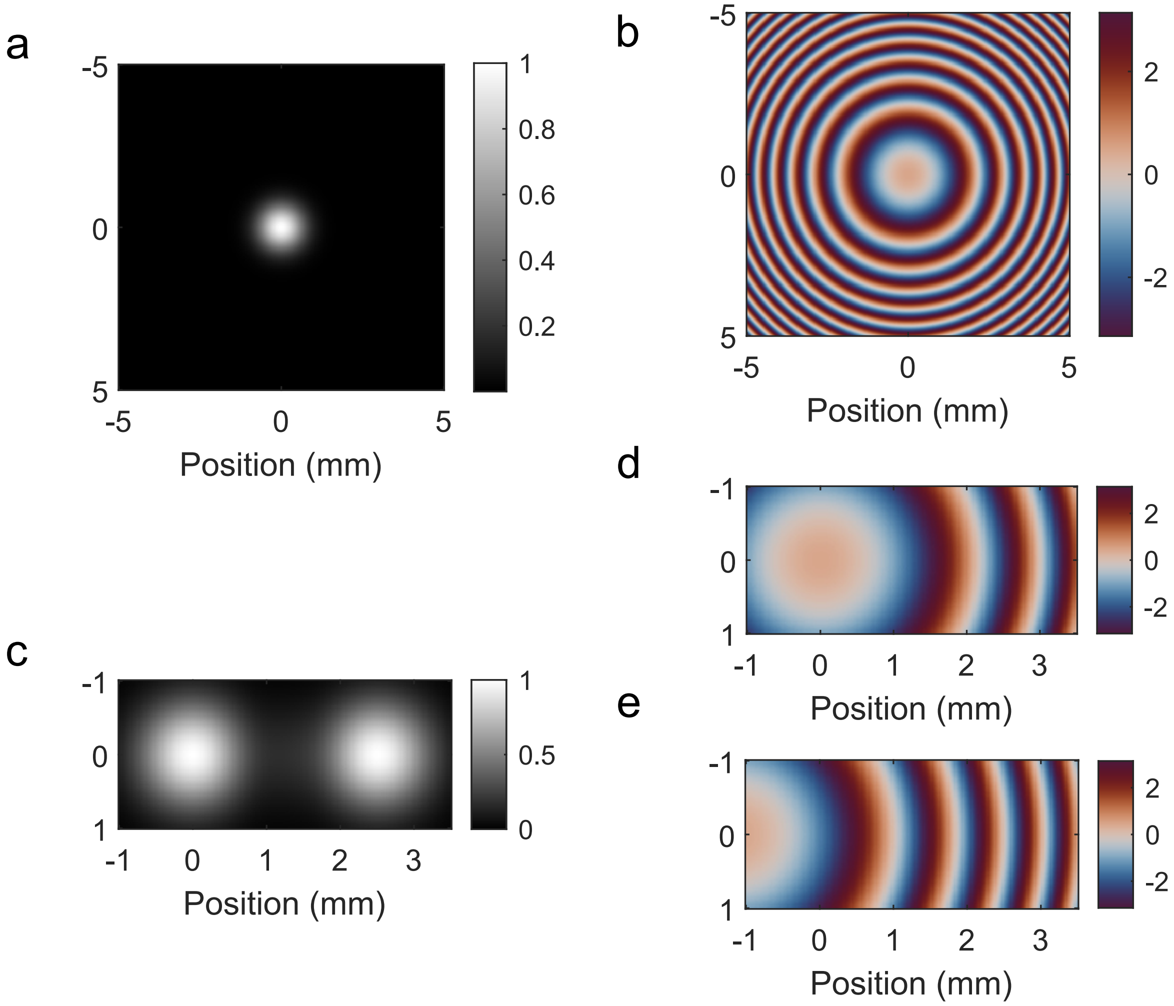

Supplementary Figure 15: Diffraction from a point source through Gaussian apertures. Calculated (a) amplitude and (b) phase at the PD plane for parameters used in scaled-up model of our approach. Amplitude (c) and phase (d,e) resulting from pairs of adjacent Gaussian apertures illuminated by a point source.

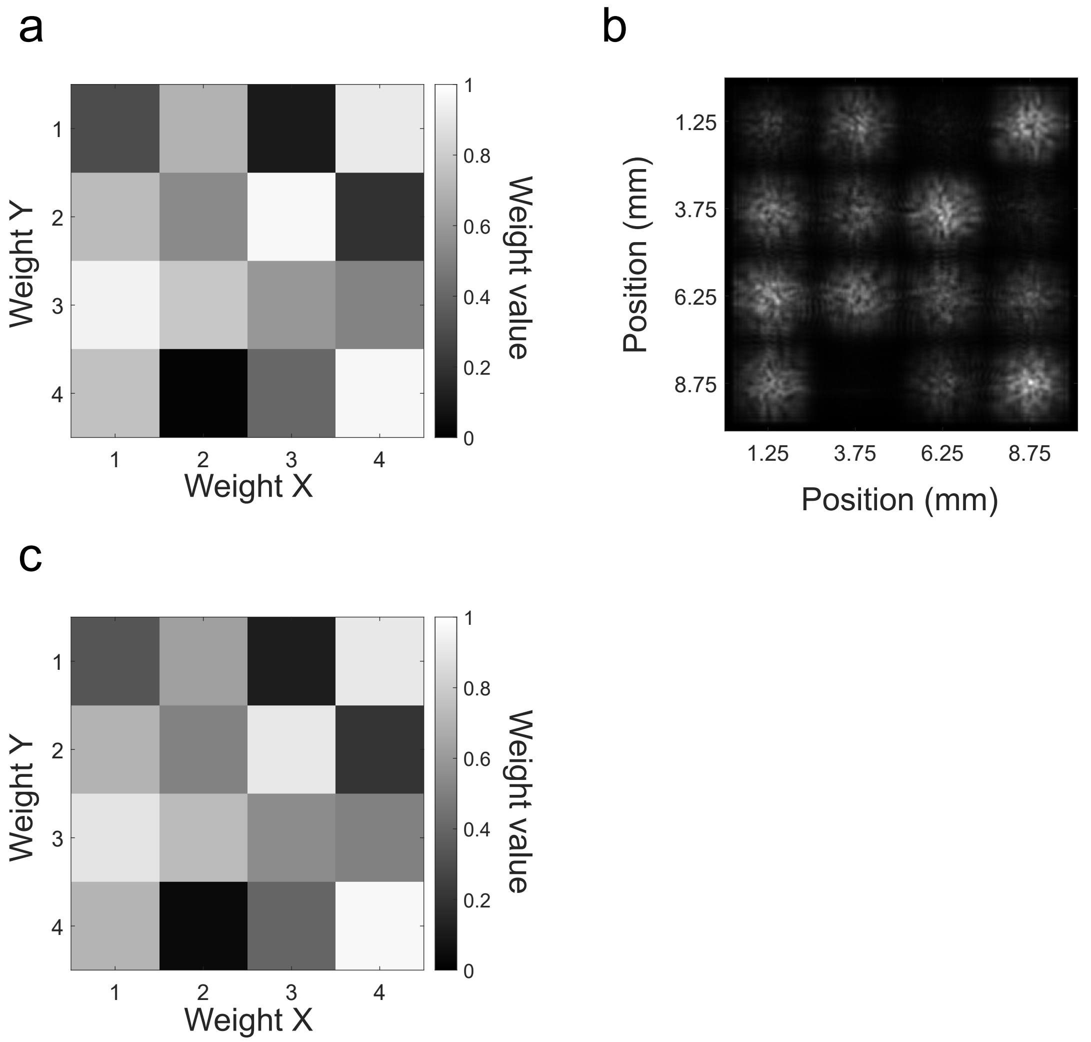

Supplementary Figure 16: Modified angular spectrum propagation predictions for LED light propagation through amplitude weights with $2.5mm$ PD spacing and $200\mu m$ LED die size. (a) Target weights in amplitude mask. (b) Light intensity at output plane. (c) Binned output values of center regions from (b).

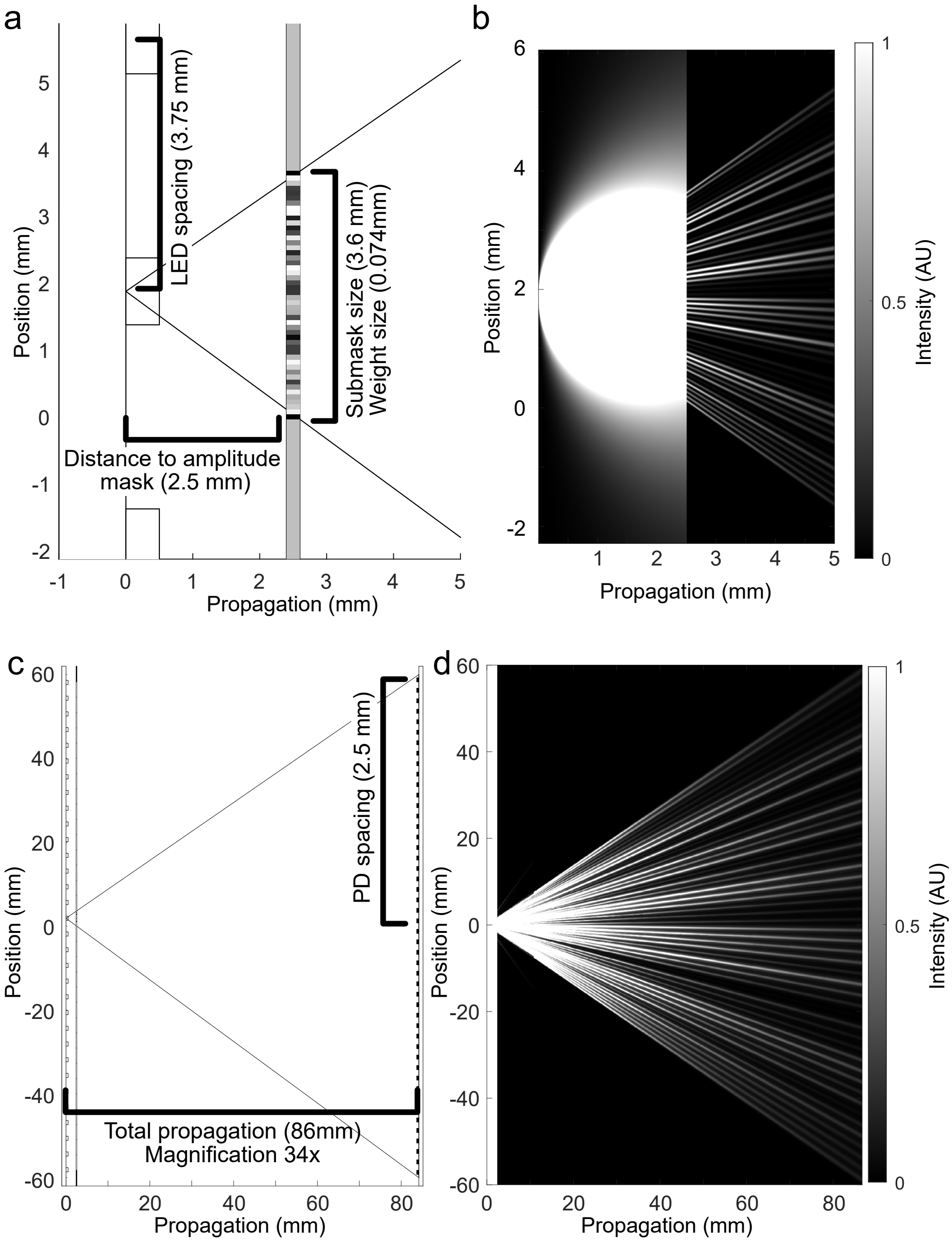

Supplementary Figure 17: Modified angular spectrum propagation calculation for scaled-up optical model. (a) In the scaled-up model, LEDs from a 32×32 rectangular array with 3.75mm spacing propagate 2.5mm to an amplitude mask. Each LED is associated with a 3.6mm submask that encodes 48×48 weights. The weights have a spacing of 0.074mm and have a Gaussian amplitude profile with spread 0.025mm to minimize diffraction effects upon propagation to the photodiode plane. (b) Sideview slice shows an example propagation from an LED through the amplitude mask. The optical parameters were chosen to minimize crosstalk between adjacent weights, even for weights at large angles relative to the LED. The white region to the left of the amplitude mask is due to saturation of the colormap and indicates the angular spread of an unobstructed LED as a function of position. (c) The total propagation distance is 84mm, resulting in an overall $M = 34$ magnification factor. The photodiode spacing of 2.5mm results in an overall detection board size that matches the LED array. (d) In the output plane, the intensity distribution is convolved with a square of size Mw_{LED}, of the magnification times the LED die size.

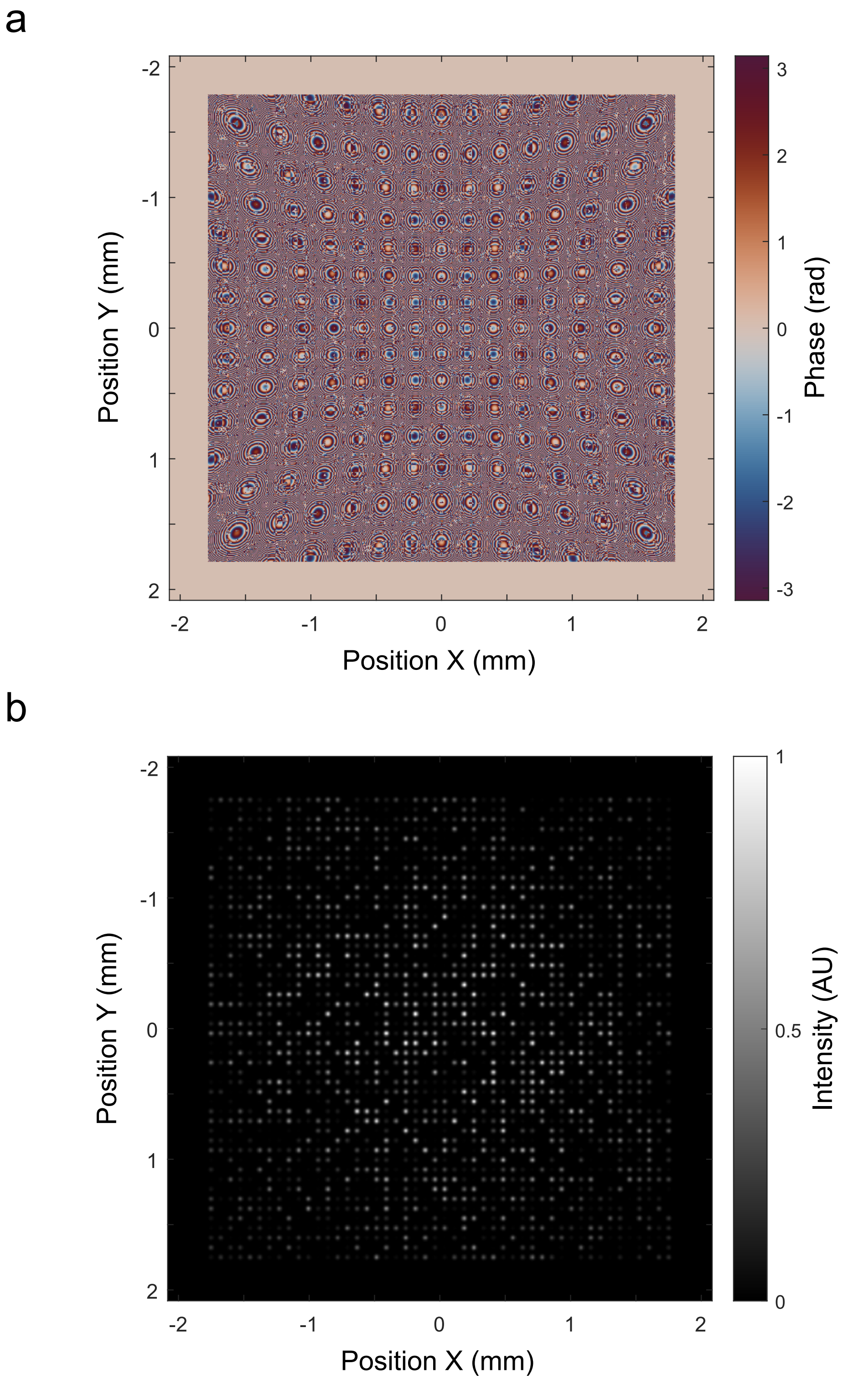

Supplementary Figure 18: Modified angular spectrum propagation field of scaled-up model showing the phase (a) and intensity (b) of the optical field from a point source immediately after propagating through the amplitude mask.

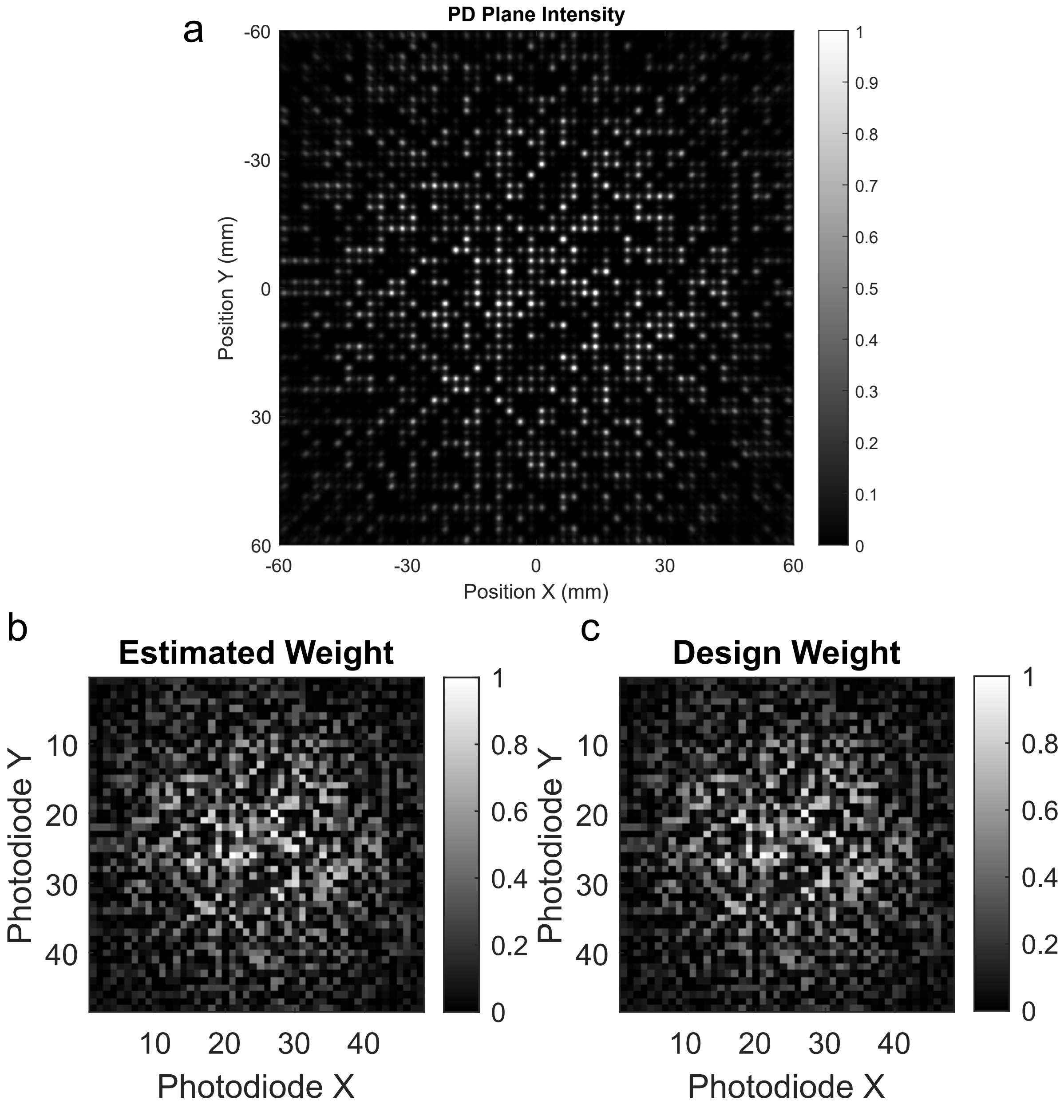

Supplementary Figure 19: Modified angular spectrum propagation predictions for LED light propagation through amplitude weights with $2.5mm$ PD spacing and $10\mu m$ LED die size. (a) Light intensity at PD plane for a 48x48 array of photodiodes. (b) Binned output values of center regions from (a). (c) Designed weights for each of the photodiode positions.

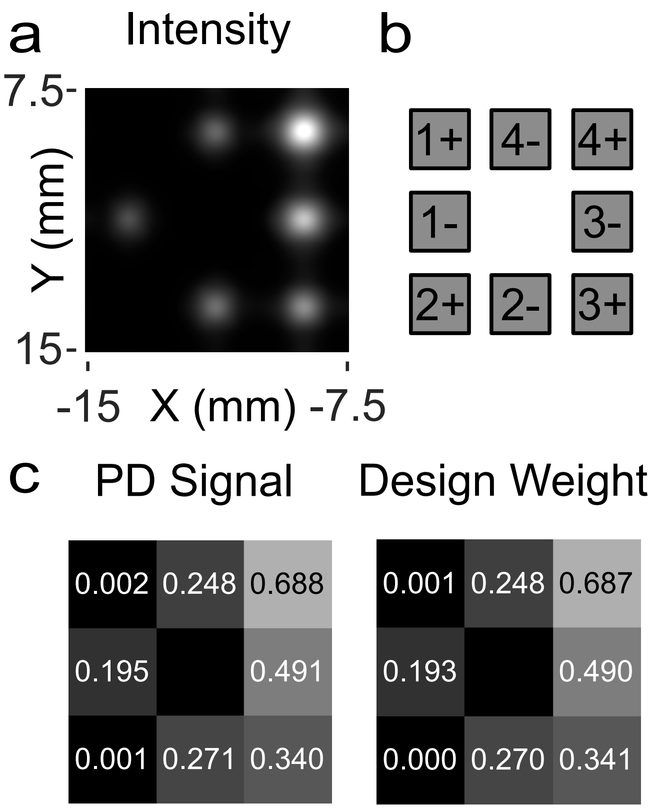

d

PD Pair	Weight +	Weight -	Difference
1	0.002	0.195	-0.193
2	0.000	0.270	-0.270
3	0.340	0.491	-0.151
4	0.688	0.248	0.440

Supplementary Figure 20: Photodiode arrangement for a 4 neuron subunit of the 32x32 LED to 48x48 photodiode scaled-up model. (a) Example 3x3 PD plane intensity from FIG. 6a,b. (b) Arrangement of positive and negative contributions to each of 4 neurons. (c) Calculated and designed weights for region in (a). (d) Effective weights after difference operation for each neuron.

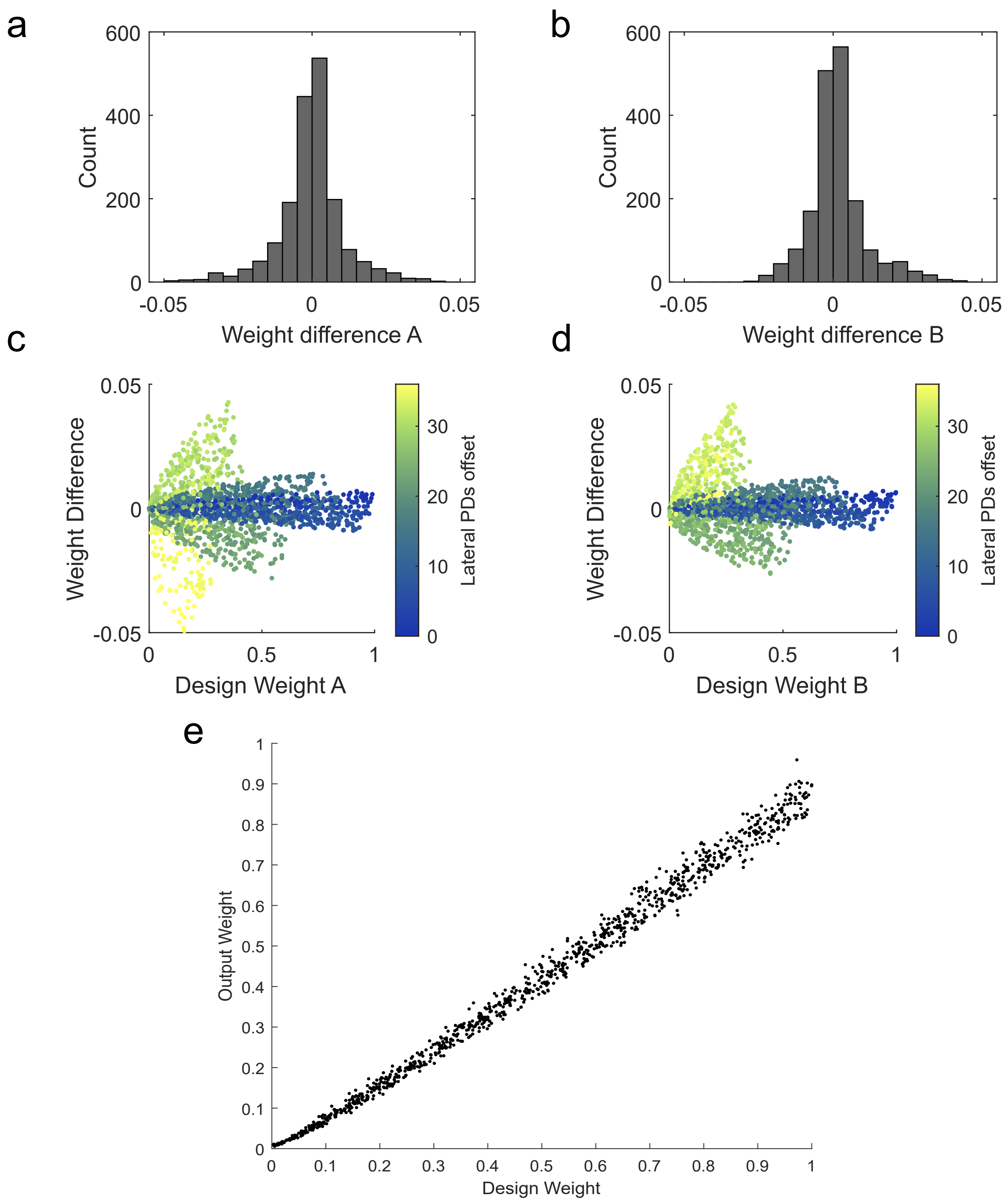

Supplementary Figure 21: Crosstalk between off-axis pairs of Gaussian weights at photodiode plane from a point source in scaled-up model. (a,b) Distribution of difference between design weight and calculated weight for random pairs of adjacent weights across the PD plane. (c,d) Data from (a,b) color-coded as a function of total lateral displacement distance from the point source position. (e) Scatterplot of simulated weight to designed weight for a photodiode weight positioned at corner of PD plane, corresponding to conditions with maximum optical crosstalk. Simulations were calculated using Rayleigh-Sommerfeld diffraction and included a 3x3 array of randomized adjacent Gaussian weights.

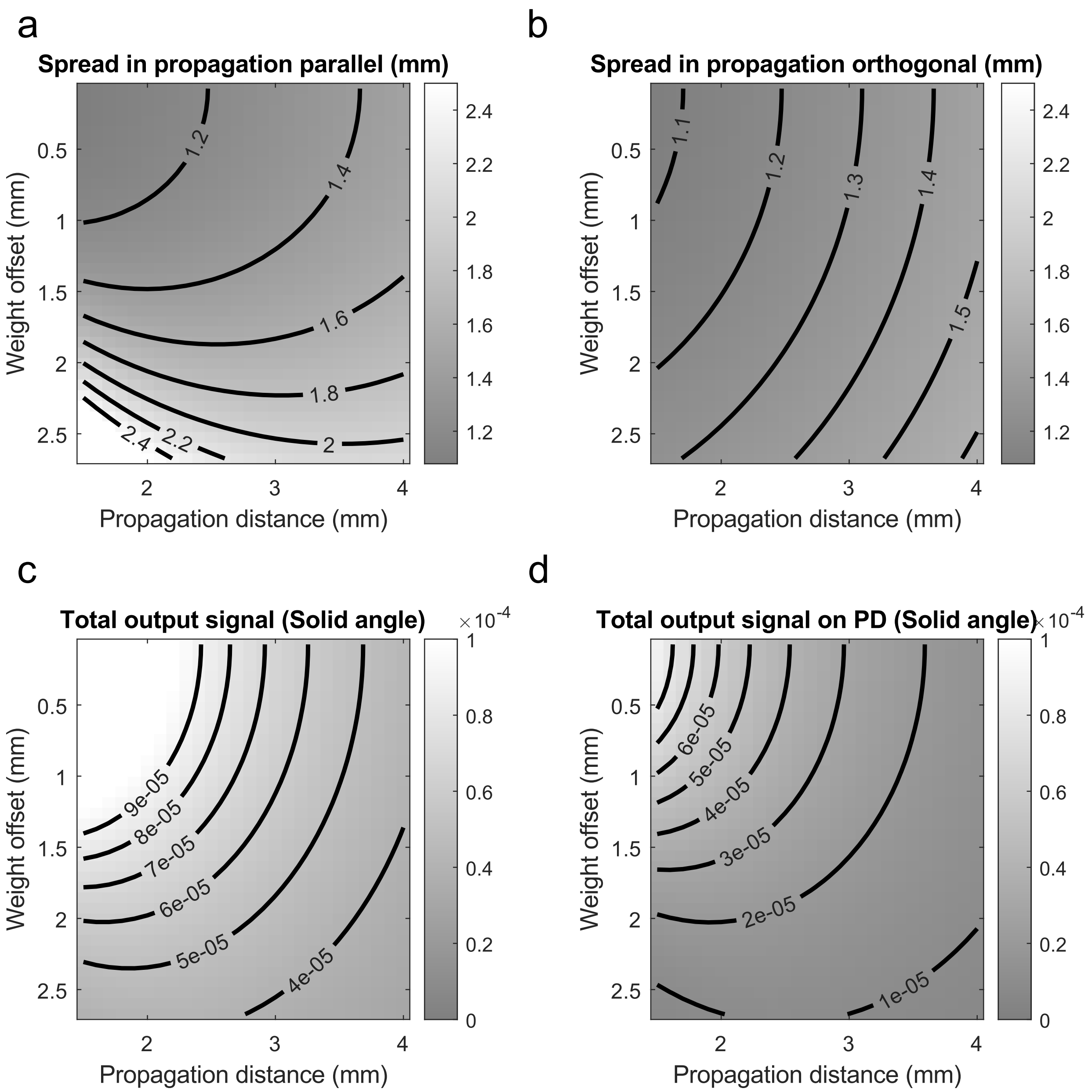

Supplementary Figure 22: Change in spread (a,b) and total integrated signal (c,d) at PD plane as a function of propagation distance and off-axis position. (a) Spread in the direction parallel to the lateral axis from point source position to Gaussian aperture. (b) Spread along the lateral axis orthogonal to (a). (c) Total solid angle per mm^2 of emitted light propagating through Gaussian aperture. (d) Total solid angle mm^2 collected on the target PD.

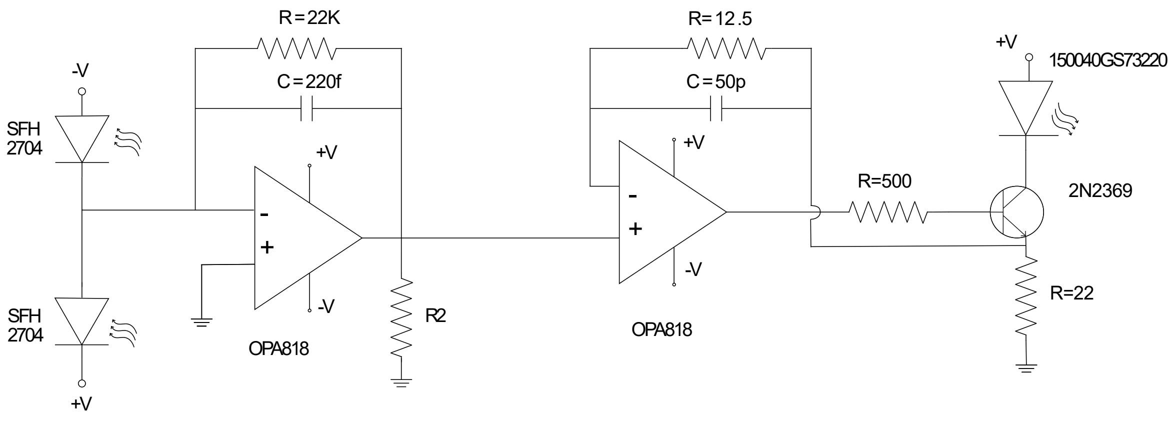

Supplementary Figure 23: Circuit model used to demonstrate electronic layer operation at 10MHz.

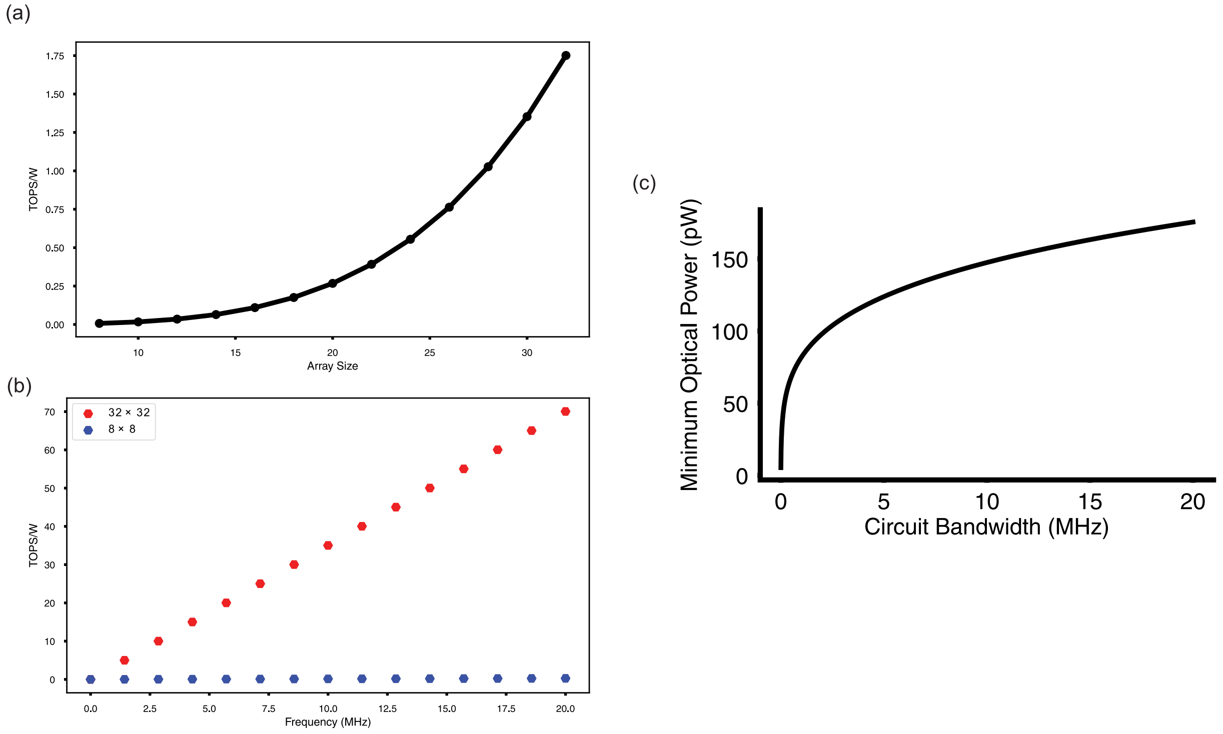

Supplementary Figure 24: (a) Scaling of system performance as a function of the photodiode array size in tera-operations per second (TOPS) per watt. (b) Scaling of accelerator as the frequency of operation is varied (c) Minimum usable optical power in the system with varying bandwidth.

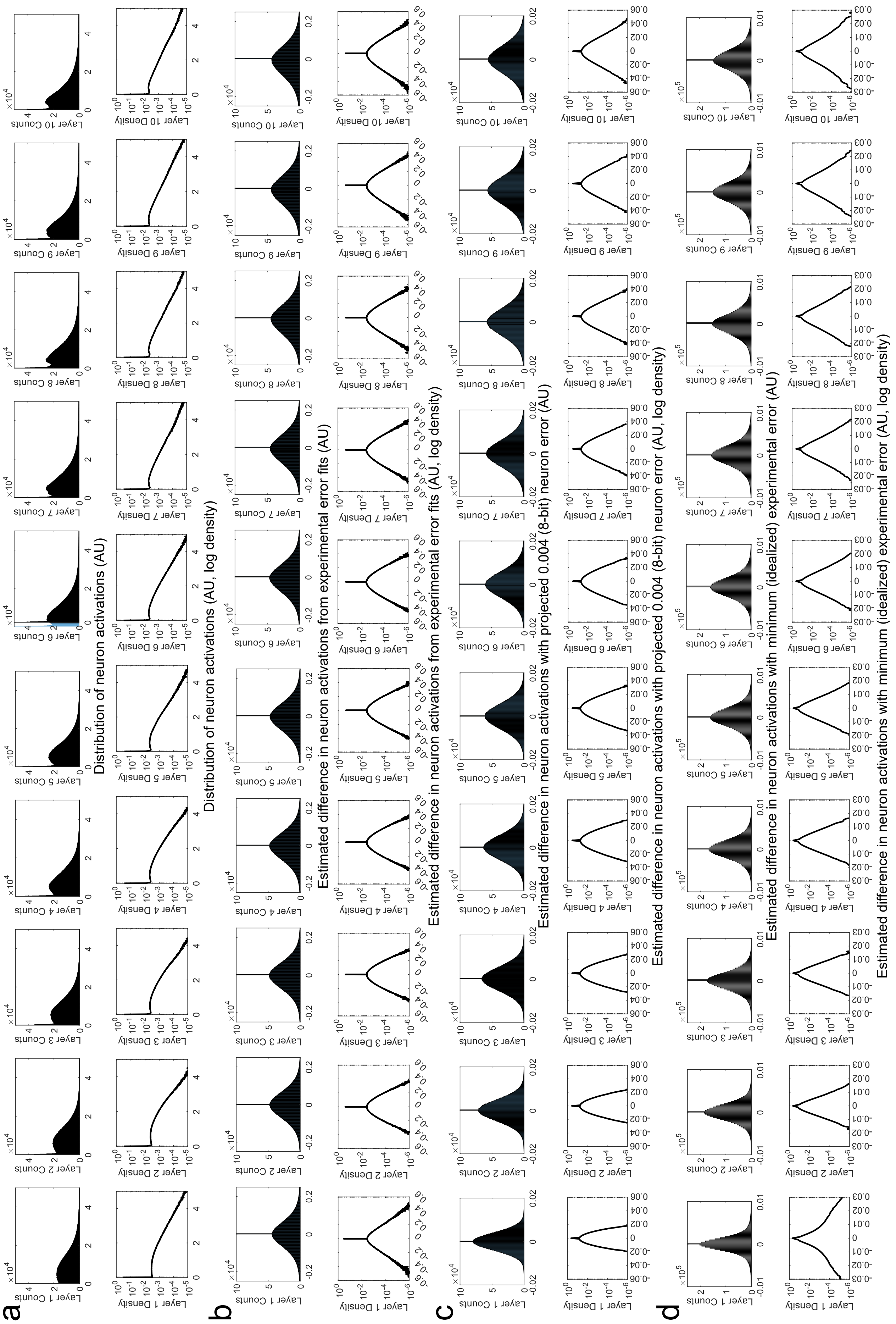

Supplementary Figure 25: (Caption next page.)

Supplementary Figure 25: Simulated error accumulation through multiple optoelectronic layers with randomized inputs and weights. Distributions after number of cumulative optoelectronic layers increase from left to right. From top to bottom, distribution are: (a) neuron activations in each layer in the absence of noise (b) difference in neuron activations with linear weighting and difference ReLU model fit errors (c) difference in neuron activations with 8-bit output error (d) difference in neuron activation with minimum error from experimental noise. For all figure parts, linear histograms (black, zero counts truncated) and log-linear density plots (white lines, zero counts not truncated) are provided.

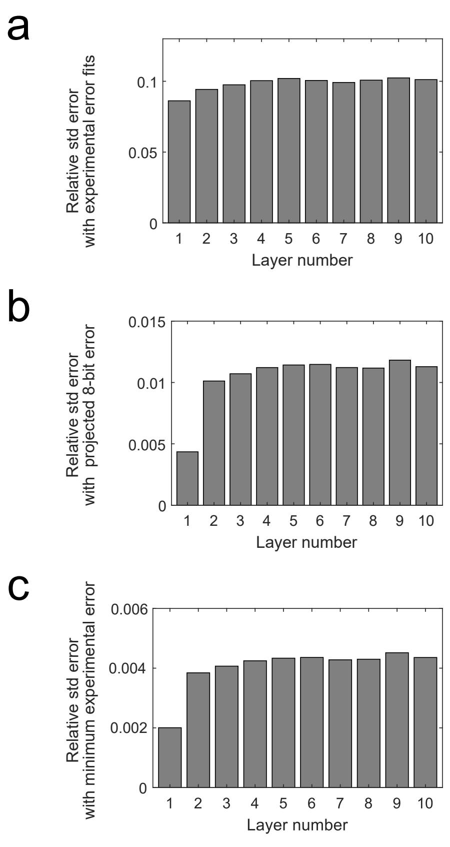

Supplementary Figure 26: Relative standard deviation of difference in neuron activation to standard deviation of neuron activation with (a) linear weighting and difference ReLU model fit errors (b) 8-bit output error (c) minimum error from experimental noise. The ratio of standard deviations compares the spread of error to the realized range of values in neuron activations.

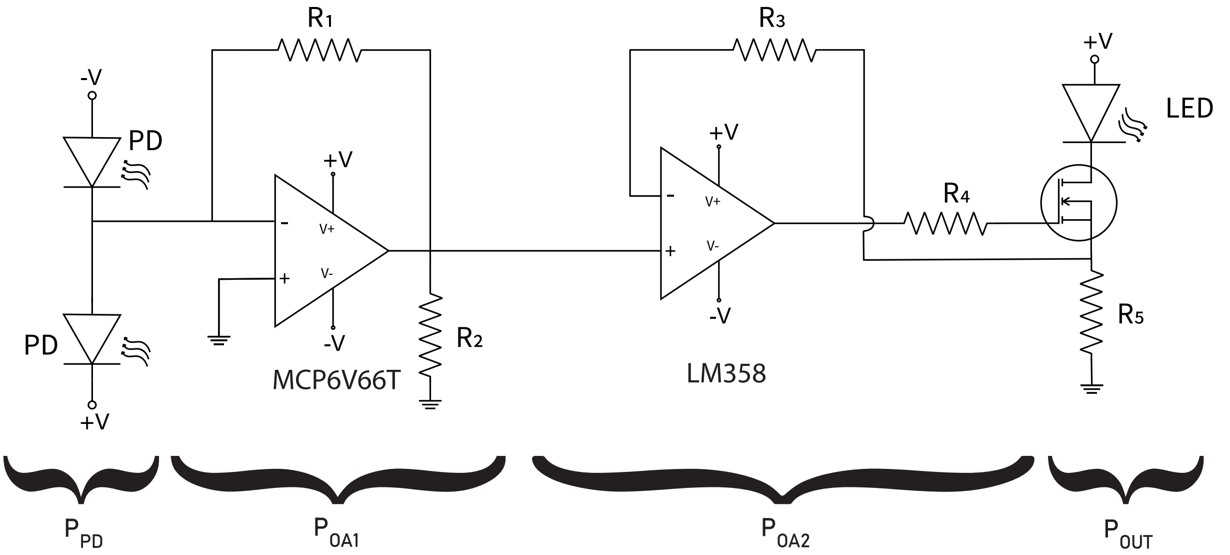

Supplementary Figure 27: Different components to the power draw in an intermediate circuit. The power draw can be represented as a combination of power drawn for biasing the photodiode, power drawn from the two operational amplifier stages and the power drawn by the LED.

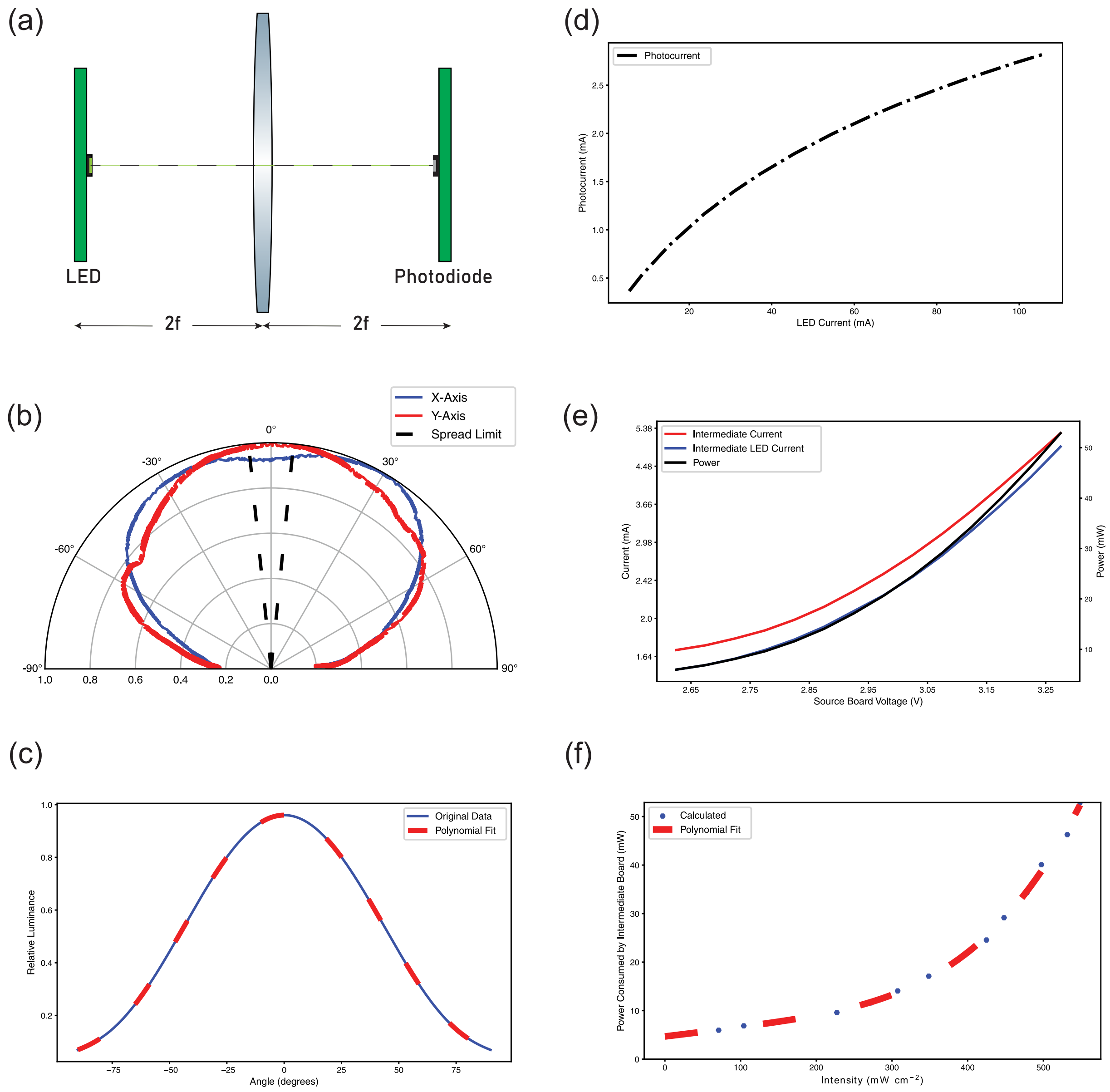

Supplementary Figure 28: Process of calculating layer efficiency. (a) Schematic of setup used for tests. (b) Angular spread of the signal from the LED (red) and the percentage of the light emitted by the LED that is collected by lens (black). (c) Directional properties of the photodiode which are applied onto the detected signal. (d) Photocurrent generated by PD. (e) Power consumption calculated for the test cases using the current drawn by the different parts of the circuit. (f) Power drawn by the circuit as a function of incident light intensity.

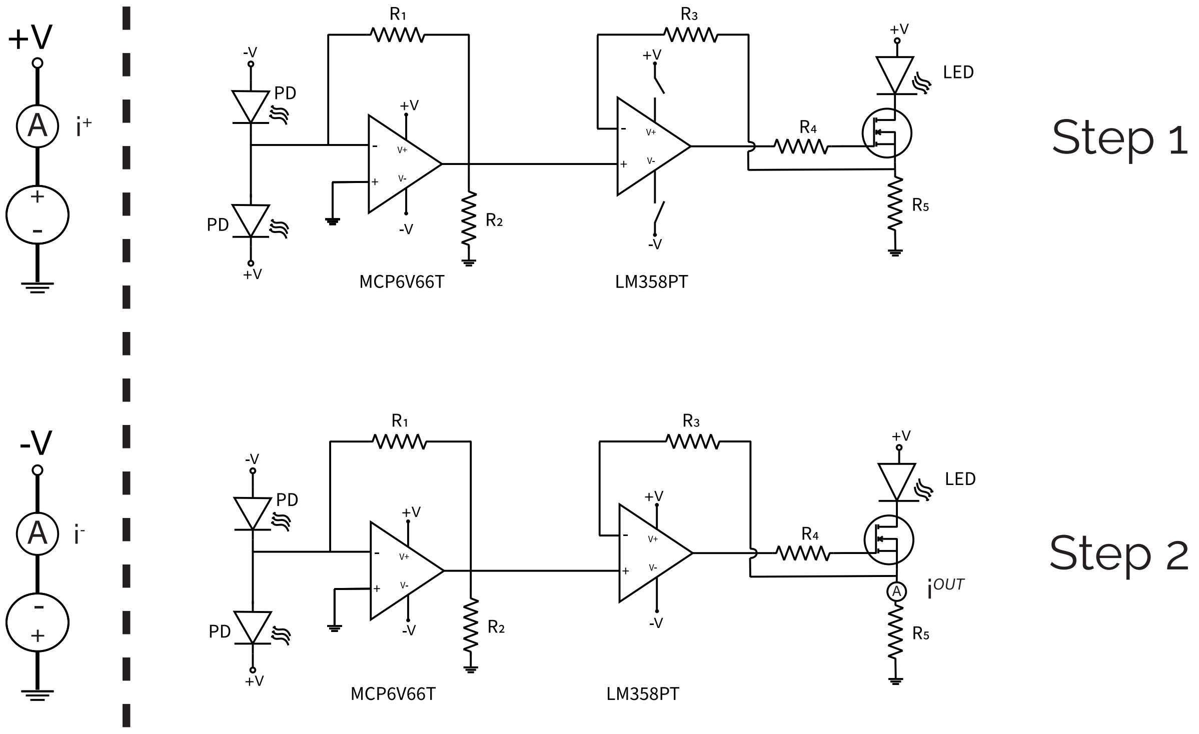

Supplementary Figure 29: Stages of measuring the power draw for the individual stages of the operational amplifier circuit. In the first step, the supply to the operational amplifier (LM358) is disconnected and the power draw from the supply is measured, followed by measuring the power drawn by the entire circuit. As a result, we can calculate the power drawn by individual stages.

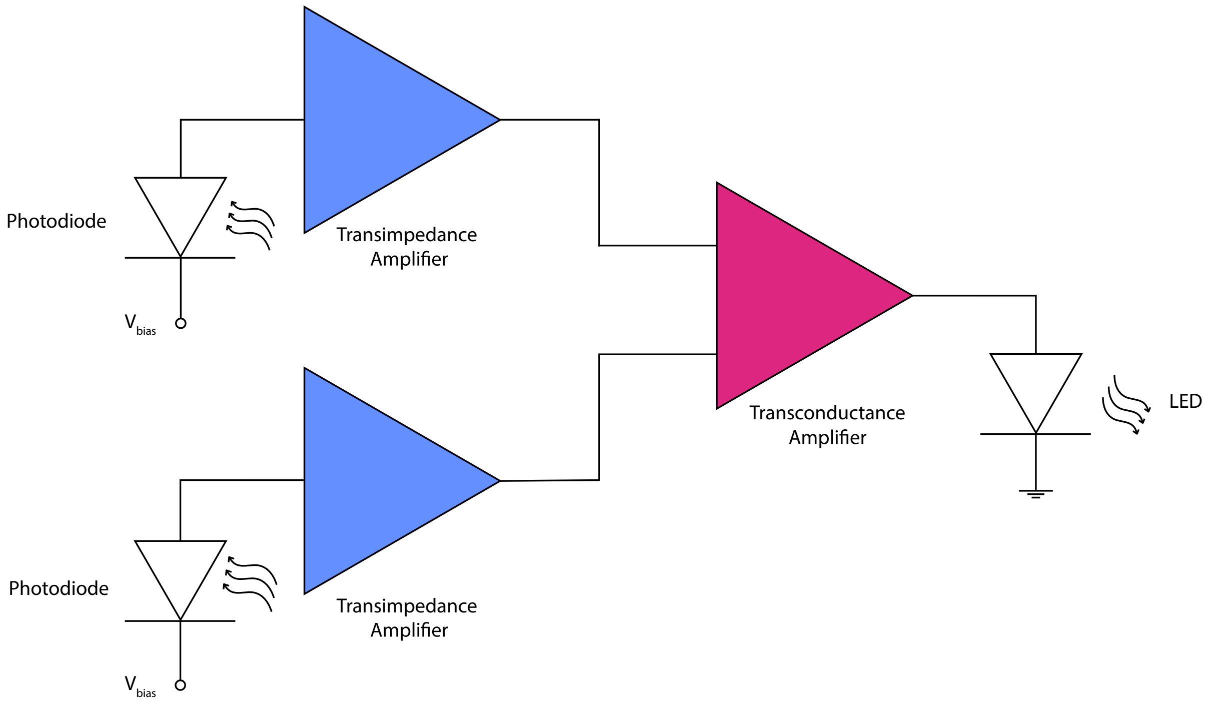

Supplementary Figure 30: Schematic of possible ASIC implementation of scaled-up model using a pair of transimpedance amplifiers (blue) and a differential transconductance amplifier (red) to drive an LED.

References

[1] XGA Data Projector LCD Product Line. *Sony.* `https://www.alldatasheet.com/datasheet-pdf/pdf/170126/SONY/LCX029CNT.html`

[2] G. Naveen I, C. Ramachandra A, & T. Manohara H. *A Low Power High-Speed 12-Bit SAR ADC using Split Capacitor Based DAC at 45 nm CMOS Technology* in *2022 IEEE 2nd Mysore Sub Section International Conference (MysuruCon)* (2022), 1-7.

[3] Li, P. *et al.* Very high external quantum efficiency and wall-plug efficiency 527 nm InGaN green LEDs by MOCVD. *Optics Express* **26**, 33108–33115 (2018).

[4] Hwang, D., Mughal, A., Pynn, C. D., Nakamura, S. & DenBaars, S. P. Sustained high external quantum efficiency in ultrasmall blue III-nitride micro-LEDs. *Applied Physics Express* **10**, 032101 (2017).

[5] Sosa, C. How to use power scaling to maximize power savings in a SAR ADC system. *Analog Design J.* 1–4 (2018).

[6] Di Patrizio Stanchieri, G., De Marcellis, A., Battisti, G., Faccio, M., Palange, E. & Guler, U. A 1.8 V low-power low-noise high tunable gain TIA for CMOS integrated optoelectronic biomedical applications. *Electronics* **11**, 1271 (2022).

[7] Khateb, F., Kaçar, F., Khatib, N. & Kubánek, D. High-precision differential-input buffered and external transconductance amplifier for low-voltage low-power applications. *Circuits, Systems, and Signal Processing* **32**, 453–476 (2013).
